# *De novo* Vessel Formation Through Cross-Talk of Blood-Derived Cells and Mesenchymal Stromal Cells in the Absence of Pre-existing Vascular Structures

**DOI:** 10.3389/fbioe.2020.602210

**Published:** 2020-11-16

**Authors:** Beate M. Rüger, Tanja Buchacher, Eva-Maria Dauber, Markus Pasztorek, Pavel Uhrin, Michael B. Fischer, Johannes M. Breuss, Gerda C. Leitner

**Affiliations:** ^1^Department of Blood Group Serology and Transfusion Medicine, Medical University of Vienna, Vienna, Austria; ^2^Turku Bioscience Centre, University of Turku and Åbo Akademi University, Turku, Finland; ^3^Department of Health Sciences, Medicine and Research, Faculty of Health and Medicine, Danube University Krems, Krems an der Donau, Austria; ^4^Department of Vascular Biology and Thrombosis Research, Center for Physiology and Pharmacology, Medical University of Vienna, Vienna, Austria

**Keywords:** vascular niche model, 3D fibrin matrix, mesenchymal stromal cells, endothelial progenitor cells, vasculogenesis, self-organization, inflammation, cell-in-cell

## Abstract

**Background:**

The generation of functional blood vessels remains a key challenge for regenerative medicine. Optimized *in vitro* culture set-ups mimicking the *in vivo* perivascular niche environment during tissue repair may provide information about the biological function and contribution of progenitor cells to postnatal vasculogenesis, thereby enhancing their therapeutic potential.

**Aim:**

We established a fibrin-based xeno-free human 3D *in vitro* vascular niche model to study the interaction of mesenchymal stromal cells (MSC) with peripheral blood mononuclear cells (PBMC) including circulating progenitor cells in the absence of endothelial cells (EC), and to investigate the contribution of this cross-talk to neo-vessel formation.

**Materials and Methods:**

Bone marrow-derived MSC were co-cultured with whole PBMC, enriched monocytes (Mo), enriched T cells, and Mo together with T cells, respectively, obtained from leukocyte reduction chambers generated during the process of single-donor platelet apheresis. Cells were embedded in 3D fibrin matrices, using exclusively human-derived culture components without external growth factors. Cytokine secretion was analyzed in supernatants of 3D cultures by cytokine array, vascular endothelial growth factor (VEGF) secretion was quantified by ELISA. Cellular and structural re-arrangements were characterized by immunofluorescence and confocal laser-scanning microscopy of topographically intact 3D fibrin gels.

**Results:**

3D co-cultures of MSC with PBMC, and enriched Mo together with enriched T cells, respectively, generated, within 2 weeks, complex CD31^+^/CD34^+^ vascular structures, surrounded by basement membrane collagen type-IV^+^ cells and matrix, in association with increased VEGF secretion. PBMC contained CD31^+^CD34^+^CD45^dim^CD14^–^ progenitor-type cells, and EC of neo-vessels were PBMC-derived. Vascular structures showed intraluminal CD45^+^ cells that underwent apoptosis thereby creating a lumen. Cross-talk of MSC with enriched Mo provided a pro-angiogenic paracrine environment. MSC co-cultured with enriched T cells formed “cell-in-cell” structures generated through internalization of T cells by CD31^+^CD45^*dim*⁣/^^–^ cells. No vascular structures were detected in co-cultures of MSC with either Mo or T cells.

**Conclusion:**

Our xeno-free 3D *in vitro* vascular niche model demonstrates that a complex synergistic network of cellular, extracellular and paracrine cross-talk can contribute to *de novo* vascular development through self-organization via co-operation of immune cells with blood-derived progenitor cells and MSC, and thereby may open a new perspective for advanced vascular tissue engineering in regenerative medicine.

## Introduction

The formation of new blood vessels is essential for normal physiological processes, and plays a key role in the repair of injured tissues. Neo-vessels are generated by sprouting of existing vascular structures through angiogenesis supported by incorporation of endothelial progenitor cells (EPC) by vasculogenesis (Asahara et al., 1999; Ribatti et al., 2001). This co-operative process takes place within a tightly controlled inflammatory microenvironment that orchestrates successful regeneration and healing. If not properly coordinated and persisting, a continuous repair process promotes excessive neovascularization and influx of more inflammatory cells eventually causing fibrosis and loss of tissue and organ function. The regenerative vascular niche environment is created via injury-induced increased vascular permeability and perivascular fibrin deposition that attract various types of leukocytes and progenitor cells, providing an ideal platform for complex interactions with resident and recruited mesenchymal stromal cells (MSC). MSC are multipotential cells found in nearly all tissues of the body where they reside close to blood vessels (Crisan et al., 2008). Upon tissue injury, they become activated through inflammatory cytokines such as interleukin (IL)-1β, tumor necrosis factor (TNF)-α, and interferon (IFN)-γ released by inflammatory cells after recruitment to the site of damage (Singer and Caplan, 2011). MSC contribute to repair processes by regulating the local immune response and secreting paracrine factors thereby establishing a regenerative environment and promoting the formation of new blood vessels (Caplan and Correa, 2011). We have recently shown that vascular endothelial growth factor (VEGF) secretion by MSC increases considerably when they encounter and have direct contact with peripheral blood-derived mononuclear cells (PBMC) in a 3D fibrin environment (Rüger et al., 2018).

Macrophages amongst leukocyte subsets, play important roles during all stages of tissue repair. Although they are mainly known as scavenger cells that phagocytize cellular debris, neutrophils, and other apoptotic cells following tissue injury (Peiser et al., 2002), macrophages also exhibit more complex roles in tissue repair (Wynn et al., 2013). They are involved in the initial cellular response following injury by secretion of various cytokines, chemokines, matrix metalloproteinases, as well as other inflammatory mediators (Wynn and Barron, 2010). Similarly, macrophages promote cellular proliferation and blood vessel development in a paracrine fashion through production of numerous growth factors including platelet derived growth factor (PDGF), transforming growth factor (TGF)-β1, insulin-like growth factor (IGF)-1, and VEGF (Shimokado et al., 1985; Rappolee et al., 1988; Berse et al., 1992; Chujo et al., 2009; Willenborg et al., 2012). Macrophages not only regulate the proliferation and expansion of neighboring parenchymal and stromal cells, but can also activate local and recruited progenitor cell populations in the niche that participate in repair. While the contribution of macrophages in tissue repair is well studied, the role of T lymphocytes in this process is not fully understood. Studies in animal models suggest that altered T cell infiltration into the wound site is associated with impaired wound healing (Swift et al., 2001), and there is evidence that CD4^+^ T cells may play a positive role in wound healing while CD8^+^ T cells may inhibit the wound healing process (Park and Barbul, 2004). Scarless skin healing has been reported in athymic nude-nu mice that are deficient in both T- and B-cells (Gawronska-Kozak et al., 2006), and a recent study using severe combined immunodeficient (SCID) mice in a wound healing model has shown that the presence of CD4^+^ T lymphocytes prevents dermal scarring by regulating inflammation and improving neovascularization (Wang et al., 2019). Regulatory T cells (Treg) promote wound healing through attenuating wound-associated inflammation (Nosbaum et al., 2016), and the generation and activation of Treg cells can be induced by MSC (Caplan, 2009; González et al., 2009; Griffin et al., 2010).

Endothelial progenitor cells play an important supportive role in postnatal angiogenesis, and are mobilized as part of the inflammatory response to injured tissues (Kopp et al., 2006). EPC have been shown to improve neovascularization in multiple injury models including wound healing (Crosby Jeffrey et al., 2000; Murohara et al., 2000; Asahara et al., 2011), but also contribute to excessive neo-vessel formation by *in situ* vasculogenesis in inflamed synovial tissues (Ruger et al., 2004). Different subtypes of circulating progenitor cells have been described and may contribute to neo-vessel formation in different ways. They include culture-derived myeloid angiogenic cells of the hematopoietic lineage, also called early outgrowth EPC, that promote angiogenesis through paracrine mechanisms, but do not give rise to mature endothelial cells (EC) (Asahara et al., 2011; Medina et al., 2011, 2017; Mund et al., 2012), and non-hematopoietic endothelial colony forming cells (ECFC), or late outgrowth EPC, that can differentiate into mature EC (Medina et al., 2017). The origin of these “true” EPC is still elusive, and they appear to be an extremely rare population within circulating blood, as *ex vivo* culture is necessary for their identification (Lin et al., 2000; Ingram et al., 2004). Interestingly, T cells seem to play an important role in the generation of both myeloid angiogenic cells and ECFC. Angiogenic T cells expressing CD3, CD31, and CXCR4 are required for colony formation and differentiation of early EPC (Hur et al., 2007), and the generation of ECFC is also T cell-dependent (Wilde et al., 2016), demonstrating the importance of microenvironmental factors including the presence of differentiated cells in the niche.

Mimicking the cellular and structural complexity of the *in vivo* vascular niche is still a challenge in the field of tissue engineering. The present study aimed to set up an *in vitro* culture environment that combines key cellular players in a biocompatible extracellular matrix simulating *in vivo* tissue repair in order to expand our current knowledge about regenerative processes and advance vascular tissue engineering for therapeutic application. The rationale behind the experimental design was based on the presence of progenitor cells with potent intrinsic angiogenic capacity in peripheral blood that are recruited to sites of injury together with inflammatory cells (e.g., Mo, T cells). Here we addressed the question whether progenitor cells and differentiated mononuclear cells in concert with MSC can form a niche environment promoting tissue repair including the formation of new vascular structures. Fibrin acts as biomimetic scaffold supporting the construction and composition of the niche environment by inducing both differentiation and stem cell marker expression of human EPC (Barsotti et al., 2011). Therefore, we established a fibrin-based xeno-free human 3D *in vitro* model using exclusively human-derived reagents and materials to study the cross-talk of MSC with PBMC obtained from leukocyte reduction system (LRS) chambers generated during the process of single-donor platelet apheresis. It has been reported that PBMC obtained from LRS chambers contain increased numbers of viable CD34^+^ progenitor cells suitable for culture (Néron et al., 2007). Using this 3D co-culture system, we investigated the vasculogenic potential through self-organization in the absence of externally added growth factors and mature EC, and analyzed the paracrine signaling signature resulting from interaction of enriched Mo and/or enriched T cells with MSC in the 3D fibrin niche environment.

## Materials and Methods

### Ethics Statement

The local Ethics Committee at the Medical University of Vienna approved the use of human bone marrow MSC (EK1193/2015) and human PBMC (EK1168/2015) in order to perform this study. All donors provided written informed consent.

### MSC and PBMC

Mesenchymal stromal cells were isolated from human bone marrow (BM) and bone fragments obtained during hip-replacement surgery and expanded in complete αMEM medium (Invitrogen, Carlsbad, CA, United States) containing 10% fetal bovine serum (GE Healthcare Life Sciences, Marlborough, MA, United States), 100 U/ml penicillin, 100 μg/ml streptomycin and 250 ng/ml amphotericin B (Sigma, St. Louis, MO, United States) at 37°C (20% O_2_ and 5% CO_2_ humidified atmosphere). MSC were characterized by flow cytometry analyses using CD90FITC (Stem Cell Technologies, Cologne, Germany), CD73PE (BD, San Jose, CA, United States), CD105FITC (BD), CD31PE (BioLegend, San Diego, CA, United States), CD34PE (BD), CD45FITC (BD) and CD14PE (BD) antibodies and a FACS Canto II^TM^ instrument (BD). Cells expressed typical MSC markers, CD90, CD73, CD105, lacked expression of CD31, CD34, CD45, and CD14, and could be differentiated into adipocytes, chondrocytes and osteoblasts. For 3D culture, MSC at passage two to five were used showing no apparent functional difference in co-culture experiments.

Peripheral blood mononuclear cells were isolated from LRS chambers (Trima Accel, Version 6.0, CaridianBCT Europe, Garching, Germany), a product generated during the process of single-donor platelet apheresis from healthy donors, by density grade centrifugation. Subpopulations of PBMC, i.e., Mo and T cells, respectively, were enriched by negative selection using RosetteSep Kits (Stem Cell Technologies, Cologne, Germany). RosetteSep Kits were used for obtaining PBMC depleted from Mo and T cells, respectively (Stem Cell Technologies). The percentage of CD14^+^CD45^+^ Mo and CD3^+^CD45^+^ T cells in whole PBMC, monocyte-enriched/depleted and T cell-enriched/depleted cell fractions were determined by flow cytometry analyses using CD14FITC (BD), CD3PE (BD), and CD45APC (BioLegend). Blood-derived progenitor cells were characterized by flow cytometry analyses using combinations of the following antibodies: CD34FITC/PE (BD), CD31FITC/PE (BioLegend), CD14FITC/PE (BD), CD117PE (BioLegend), and CD45APC.

### Culture of MSC and Co-culture of MSC With Immune Cells in 3D Fibrin Matrices

Co-culture experiments were set up using MSC obtained from five donors at different passages (*n* = 10) and PBMC from individual donors (*n* = 10). Further, MSC at comparable passages, were co-cultured with individual donor-derived enriched Mo (*n* = 6), individual donor-derived enriched T cells (*n* = 9), enriched T cells together with enriched Mo obtained from separate donors (*n* = 4), individual donor-derived PBMC depleted from Mo (*n* = 6), and individual donor-derived PBMC depleted from T cells (*n* = 6), respectively. In addition and parallel to co-cultures, the same MSC samples used for co-cultures were cultivated without immune cells. 3D cultures were set up in 24-well plates (Corning, Berlin, Germany). Cells were embedded in fibrin matrices in a ratio 1:100 (for MSC:PBMC and MSC:Mo + T) and 1:50 (for MSC:Mo and MSC:T), using 5 × 10^4^ MSC/well. Fibrin matrices were prepared as described previously with minor modifications (Ruger et al., 2008; Rüger et al., 2018). In brief, human fibrinogen (2 mg/ml; Calbiochem, Darmstadt, Germany) was dissolved in PBS, human plasma thrombin (0.45 U/ml, Sigma) was added to the fibrinogen solution containing MSC and/or immune cells and gel formation occurred by incubation at 37°C for 30 min. Cells were cultured using complete αMEM medium (Invitrogen) containing 10% human AB serum (GMP grade, PAN-Biotech, Aidenbach, Germany) without externally added growth factors for up to 2 weeks. Control experiments were performed culturing Mo separated from MSC by a 0.4 μm transwell insert (Corning), as well as culturing PBMC, enriched Mo, enriched T cells, and enriched Mo together with enriched T cells, respectively, without MSC. Medium was changed every 3 days. Cellular re-arrangement was monitored using a phase contrast microscope (Olympus IMT-2, Tokyo, Japan) and documented using a digital camera (Olympus DP50).

### DNA Extraction and Analysis of Polymorphic Marker

In order to determine the origin of EC within the vascular structures that develop in the 3D fibrin gels, we performed gender-mismatched co-cultures using 6-well plates. Male MSC were co-cultured with female PBMC in 3D fibrin matrices for 1 week. Alternatively, female MSC were co-cultured with male PBMC. To obtain single cell suspensions, the fibrin gels were dissolved using nattokinase (NSK-SD; Japan Bio Science Laboratory Co., Ltd., Osaka, Japan) (Carrion et al., 2014), and DNA extracted from FACS-sorted CD34^+^CD45^–^ EC and CD34^–^CD45^+^ leukocytes with the QIAamp DNA Investigator Kit (Qiagen GmbH, Hilden, Germany). An insertion/deletion (indel) polymorphism in the X–Y homologous gene amelogenin was amplified by PCR and subjected to fragment analysis by capillary electrophoresis (CE) according to Steinlechner et al. (2002). In addition, the NGM Detect PCR amplification kit, a 16-locus multiplex PCR of highly polymorphic short tandem repeat (STR) markers was performed and analyzed on an ABI 3130 Genetic Analyzer according to the manufacturer’s instructions (Applied Biosystems by Thermo Fisher Scientific, Waltham, MA, United States).

### Confocal Laser Scanning Microscopy (CLSM) of Intact 3D Cultures

In order to perform confocal laser scanning microscopy (CLSM) of whole 3D cultures, immunofluorescence analyses of intact 3D fibrin matrices containing the self-organized structures were performed as described previously (Rüger et al., 2018), and stained fibrin gels transferred to ibidi chambers (ibidi GmbH, Martinsried, Germany) for CLSM. Briefly, fibrin matrices were fixed with 4% paraformaldehyde and incubated with a buffer solution containing 0.1% BSA, 0.2% Triton X-100, 0.05% Tween 20 in PBS followed by a blocking step with 20% normal donkey serum (Jackson Immuno Research, West Grove, PA, United States). Fibrin gels were incubated with anti-human CD31 (mouse IgG_1_, 8 μg/ml, Dako) or anti-human CD34 (mouse IgG_1_, 4 μg/ml, Cell Marque, Rocklin, CA, United States) together with rabbit anti-Col-IV (7.5 μg/ml, Novus Biologicals, Cambridge, United Kingdom) for 6 h at room temperature. The cultures were washed with buffer solution and incubated simultaneously with donkey anti-mouse IgG_1_ Alexa Fluor (AF) 488 and donkey anti-rabbit AF555 (2.6 μg/ml, Molecular Probes, Life Technologies, Carlsbad, CA, United States) and cell nuclei stained with DAPI. Omission of primary antibodies and the use of isotype-matched non-immune antibodies served as controls. For triple labeling, the 3D constructs stained with CD31 or CD34 and Col-IV antibodies were blocked with 20% mouse serum (Jackson Immuno Research) and incubated with AF647-mouse anti-CD45 (2.5 μg/ml, BioLegend) or AF647-mouse anti-CD3 (2.5 μg/ml, BioLegend). The cultures were washed with buffer solution, cell nuclei stained with DAPI, and 3D gels kept in PBS at +4°C until CLSM analyses. All 3D cultures were evaluated using a LSM 700 or LSM 780 confocal laser scanning microscope (Carl Zeiss, Jena, Germany) and the acquired images analyzed with the ZEN image processing and analysis software program (Zeiss).

### Cytokine Determination

Cell-free supernatants of 3D MSC mono-cultures and MSC co-cultured with enriched Mo or enriched T cells, and enriched Mo together with enriched T cells, respectively, were analyzed 24 h after embedding the cells within fibrin gels, using a cytokine array kit (Proteome Profiler Human XL Cytokine Array Kit, R&D Systems, Minneapolis, MN, United States). For each group, pooled samples of four experiments were used. The intensities of chemiluminescence signals were determined by subtraction of background noise, and the mean gray values of duplicate cytokine spots were determined using Bio-Rad Quantity One software (Bio-Rad, Hercules, CA, United States). VEGF secretion levels in the supernatants of separate samples were quantified utilizing a commercially available ELISA Duoset system (R&D). Supernatants of 3D co-cultures set up as described above, were collected after 24 h, on day 3 and on day 6. VEGF levels were determined in co-cultures of MSC with PBMC (*n* = 9), enriched Mo (*n* = 4), enriched T cells (*n* = 4), enriched Mo together with enriched T cells (*n* = 4), Mo-depleted PBMC (*n* = 6), and T cell-depleted PBMC (*n* = 4), respectively. In addition, supernatants of MSC mono-cultures and supernatants of immune cells cultured without MSC, set up in parallel and corresponding to each co-culture experiment, were collected at the same time points. Experiments were performed with each sample in duplicate, and data are expressed as mean values ± SD. In 3D co-cultures where MSC and Mo were physically separated by a transwell insert, VEGF levels in the supernatants were measured by ELISA (R&D) on day 6. The assays were performed according to the reference manual, and the samples were measured in technical duplicates. Optical density values were measured at 450 nm on an ELISA plate reader (anthos Mikrosysteme, Krefeld, Germany).

### Statistics

Statistical analyses were performed using the software package SPSS Statistics for Windows, version 22.0 (SPSS Inc., Chicago, IL, United States). Data were analyzed for statistical significance by unpaired *t*-test and expressed as means ± SD. Significance was concluded when a probability value (*p* value) was lower than 0.05. (ns: not significant; ^∗^ *p* ≤ 0.05; ^∗∗^ *p* ≤ 0.01; ^∗∗∗^ *p* ≤ 0.001).

## Results

### Vascular Structures Develop *de novo* During Co-culture of MSC and PBMC in 3D Fibrin Matrices

**FIGURE 1:**
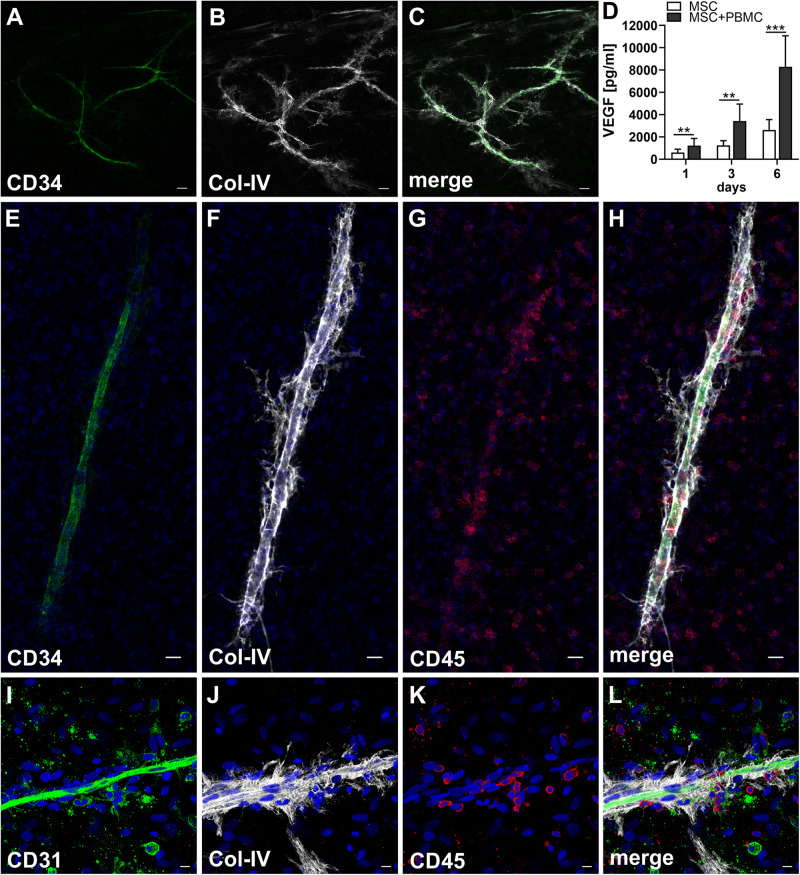
Co-culture of MSC with PBMC in 3D fibrin matrix leads to the development of vascular structures. Representative images from three individual experiments are shown. **(A–C)** Experiment 1. The cells forming vascular structures with several branch points express **(A)** CD34 and are surrounded by **(B)** Col-IV matrix. **(C)** Merge. **(A–C)** CLSM images of an intact fibrin gel on day 10. Scale bars, 50 μm. **(D)** Determination of VEGF by ELISA in cell-free supernatants of 3D MSC monocultures (*n* = 9) and corresponding MSC-PBMC co-cultures, on day 1, 3, and 6, respectively. The data are expressed as mean values ± SD. **p value ≤ 0.01, ***p value ≤ 0.001. **(E–H)** Experiment 2. **(E)** The CD34^+^ neo-vessel is surrounded by **(F)** Col-IV matrix and develops in close vicinity to **(G)** CD45^+^ leukocytes. **(H)** Merge. **(E–H)** Nuclei stained with DAPI. CLSM images of an intact fibrin gel on day 10. Scale bars 20 μm. **(I–L)** Experiment 3. Developing vascular strand expresses **(I)** CD31, **(J)** with partial co-expression of Col-IV, and is surrounded by Col-IV^+^CD31^-^ perivascular cells. **(K)** CD45^+^ leukocytes are distributed around and closely associated with the developing vascular structure. **(L)** Merge. **(I–L)** Nuclei stained with DAPI. CLSM images of an intact fibrin gel on day 10. Collapsed 21.6 μm z-stack consisting of 54 consecutive images. Scale bars 10 μm.

**FIGURE 2:**
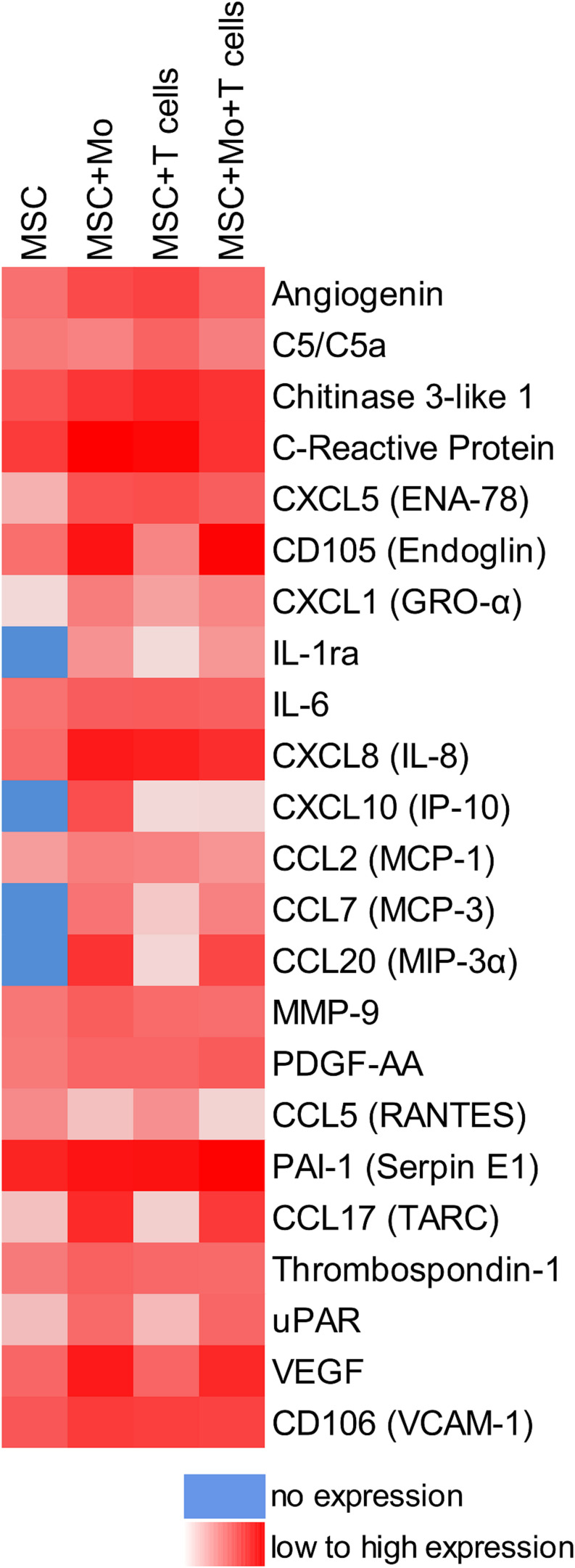
cross-talk of MSC with Mo and/or T cells in 3D fibrin matrix alters the paracrine niche environment. Heat map reflecting the secretion profile by MSC cultured for 24 h in 3D fibrin matrix compared to secretion profile by MSC co-cultured with enriched Mo, enriched T cells, and enriched Mo together with enriched T cells, respectively. Pooled samples from four individual experiments.

When MSC and PBMC were co-cultured in a xeno-free niche environment, complex vascular structures with several branch points developed within one to 2 weeks in the originally EC-free avascular 3D fibrin gels (Figures 1A–C). EC of newly formed vascular structures expressed CD34 (Figures 1A,E) and CD31 (Figure 1I), and were surrounded by basement-membrane collagen type (Col)-IV expressing MSC and matrix (Figures 1B,F,J). CD45^+^ leukocytes were found in close vicinity and aligned to developing neo-vessels (Figures 1G,K and Supplementary Animated z-stack S1). PBMC isolated from LRS chambers contained 24.0 ± 7.6% CD14^+^ Mo, 49.7 ± 11.7% CD3^+^ T cells and 0.24 ± 0.12% cells with a distinct non-myeloid progenitor phenotype expressing CD34 and CD31, with low CD45 and no CD14 (Table 1 and Supplementary Figure S1). The majority of CD34^+^ progenitor cells also expressed CD117 (Supplementary Figure S1). No vascular structures were detected in fibrin gels with MSC only and in PBMC-mono-cultures, respectively (data not shown), and 3D PBMC cultures without support of MSC showed considerable fibrinolysis in the second week of culture (data not shown). Using a cytokine array kit we found that MSC within 3D fibrin matrices produce a number of pro-inflammatory mediators that are also involved in new vessel formation including interleukin (IL)-6, complement component C5/C5a, CCL2, CXCL8, VEGF, angiogenin, endoglin, PAI-1, thrombospondin, VCAM-1, PDGF-AA, and MMP-9 (Figure 2). VEGF release by MSC progressively increased during culture, and in co-culture with PBMC, VEGF secretion was significantly higher compared to MSC mono-cultures after 24 h, at day 3 and 6, respectively, as demonstrated by ELISA (Figure 1D and Table 2). VEGF secretion levels of PBMC cultured without MSC support were under 100 pg/ml after 24 h fibrin matrix-exposure, and were below detection level on day 6 of culture (data not shown).

**TABLE 1:** Percentage of CD14^+^ Mo, CD3^+^ T cells and CD34^+^CD31^+^CD45^dim^CD14^–^ progenitor-type cells in PBMC, Mo-enriched/depleted and T cell-enriched/depleted PBMC used for 3D culture experiments.

**Population**	**%CD14^+^**	**%CD3^+^**	**%progenitor-type**
PBMC	24.0 ± 7.6	49.7 ± 11.7	0.24 ± 0.12
Mo enriched	73.5 + 6.2	<0.1	0.76 ± 0.21
T cells enriched	0	96.4 ± 3.6	0.40 ± 0.36
Mo-depleted	<0.1	69.0 ± 12.2	0.32 ± 0.20
T cell-depleted	46.9 ± 8.1	<0.1	0.45 ± 0.29

*The data are expressed as mean values ± SD.*

**TABLE 2:** VEGF secretion levels measured in cell-free supernatants of 3D (co)-cultures on day 1, 3, and 6.

		**VEGF [pg/ml]**	

	**Day 1**	**Day 3**	**Day 6**
MSC + PBMC	1212 ± 648	3420 ± 1525	8284 ± 2790
MSC + Mo enriched	1908 ± 587	3492 ± 1299	6820 ± 907
MSC + T cells enriched	321 ± 133	672 ± 130	1589 ± 295
MSC + Mo + T cells	1694 ± 962	3785 ± 1330	6897 ± 2061
MSC + Mo-depleted	259 ± 79	950 ± 80	4444 ± 1153
MSC + T cell-depleted	1430 ± 296	3242 ± 382	8854 ± 1930

MSC only	427 ± 190	1241 ± 424	2620 ± 936

*The data are expressed as mean values ± SD.*

### EC of Neo-Vascular Structures Are Derived From Cells Present in Peripheral Blood

**FIGURE 3:**
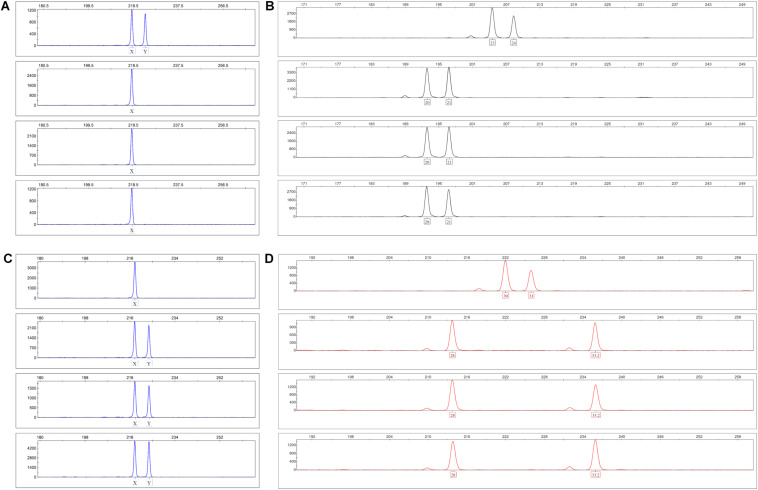
Indel and STR genotypes of post-culture FACS-sorted EC and leukocytes are identical with pre-culture PBMC in fragment analysis. **(A,B)** Male MSC were co-cultured with female PBMC in 3D fibrin matrices for 1 week. Panel **(A)** shows the X-Y homologous indel polymorphism in the amelogenin gene and panel **(B)** the STR polymorphism FGA (4q28) top down: pre-culture MSC, PBMC, post-culture FACS-sorted CD34^+^CD45^-^ EC and CD34^-^CD45^+^ leukocytes. **(C,D)** Female MSC were co-cultured with male PBMC in 3D fibrin matrices for 1 week. Panel **(C)** shows the X-Y homologous indel polymorphism in the amelogenin gene and panel **(D)** the STR polymorphism D21S11 (21q21.1) top down: pre-culture MSC, PBMC, post-culture FACS-sorted CD34^+^CD45^-^ EC and CD34^-^CD45^+^ leukocytes.

As both BM-derived EPC contribute to new vessel formation (Asahara et al., 1999), and MSC can differentiate into EC (Oswald et al., 2004; Silva Guilherme et al., 2005; Janeczek Portalska et al., 2012; Wang et al., 2018), we set up experiments to determine the origin of EC forming the neo-vessels in the 3D fibrin gels. In order to avoid problems related to potential cell toxicity and change in cellular behavior induced by labeling cells with cell tracking dyes, we performed gender-mismatched co-cultures using non-manipulated MSC and PBMC. Male MSC were co-cultured with female PBMC, and female MSC were co-cultured with male PBMC, respectively, in 3D fibrin matrices. DNA isolated from FACS-sorted fibrin gel-derived CD34^+^CD45^–^ EC and CD34^–^CD45^+^ leukocytes was subjected to analyses of indel- and STR polymorphisms by PCR and fragment analysis by capillary electrophoresis. The results showed that both the CD34^–^CD45^+^ hematopoietic cells as well as the CD34^+^CD45^–^ cells, i.e., EC of the vascular structures formed in 3D co-cultures, originated from cells circulating in the peripheral blood (Figure 3).

### Cross-Talk of MSC With Monocytes/Macrophages Creates a Pro-angiogenic Paracrine Microenvironment in 3D Fibrin Gels

**FIGURE 4:**
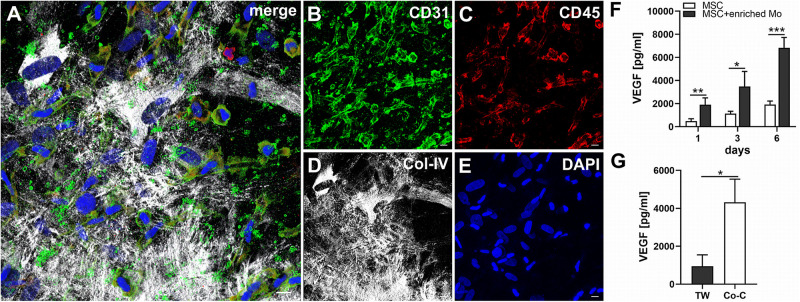
MSC cross-talk with enriched Mo increases VEGF levels in the niche environment. **(A–E)** Representative CLSM images of MSC co-cultured with enriched Mo in 3D fibrin matrix on day 14 showing numerous round and spindle-shaped cells expressing **(B)** CD31 with differential co-expression of **(C)** CD45, embedded in a meshwork of **(D)** Col-IV^+^ MSC and matrix. **(A)** Merge. **(E)** DAPI stain. Collapsed 21.15 μm z-stack consisting of 47 consecutive images. Scale bars, 10 μm. **(F)** Determination of VEGF by ELISA in cell-free supernatants of 3D MSC monocultures (*n* = 4) and corresponding co-cultures of MSC with enriched Mo, on day 1, 3, and 6, respectively. The data are expressed as mean values ± SD. *p value ≤ 0.05, **p value ≤ 0.01, ***p value ≤ 0.001. **(G)** VEGF levels in cell-free supernatants of 3D co-cultures where MSC were physically separated from enriched Mo by a transwell insert with 0.4 μm pore size (TW) and corresponding 3D co-cultures allowing for contact of MSC with enriched Mo on day 6 (*n* = 3). The data are expressed as mean values ± SD. *p value ≤ 0.05.

Next, we analyzed the contributions of Mo to vascular morphogenesis and paracrine signaling in the *in vitro* vascular niche environment. The Mo-enriched fraction contained 73.5 ± 6.2% cells expressing CD14 with <0.1% CD3^+^ cells present, and 0.76 ± 0.21% cells with the CD34^+^CD31^+^CD45^dim^CD14^–^ progenitor phenotype as determined by flow cytometry analysis (Table 1). Although Mo enrichment significantly increased the number of CD34^+^CD31^+^CD45^dim^CD14^–^ progenitor cells in comparison to whole PBMC (Supplementary Figure S1F), no vascular structures were detected when enriched Mo were co-cultured with MSC for 10–14 days in the 3D fibrin environment (Figures 4A–E). CLSM analysis of co-cultures in fibrin gels revealed the presence of numerous CD31^+^ cells (Figure 4B) co-expressing CD45 (Figure 4C) in close contact to MSC that expressed Col-IV (Figure 4D). Analysis of the cytokine secretion profile resulting from interaction of MSC with enriched Mo for 24 h in fibrin matrices revealed that several mediators involved in inflammatory neovascularization were newly detected or up-regulated in the supernatants of co-cultures when compared to MSC mono-cultures, e.g., CXCL1, CXCL5, CXCL8, CXCL10, CCL7, CCL17, CCL20, IL-1ra, and IL-6 (Figure 2). In contrast, while MSC monocultures secreted CCL5, the presence of Mo for 24 h decreased CCL5 release in co-cultures (Figure 2). Interaction of MSC with enriched Mo for 24 h in the fibrin matrix led to significantly increased VEGF secretion compared to MSC mono-cultures (Figure 4F and Table 2). VEGF levels in co-cultures progressively increased between day 1 and 6 reaching levels similar to those found in supernatants of MSC co-cultured with whole PBMC (MSC + Mo, day 6: 6820 ± 907, MSC + PBMC, day 6: 8284 ± 2790) (Figure 4F and Table 2). 3D transwell experiments using 0.4 μm inserts to separate MSC from enriched Mo revealed that the high VEGF levels were dependent on direct contact of MSC with Mo (Figure 4G). When PBMC were depleted from CD3^+^ T cells the percentage of Mo increased around 2-fold compared to whole PBMC samples to 46.9 ± 8.1% (Table 1). Accordingly, VEGF levels measured in the supernatants of these co-cultures on day 1 and 3, respectively, were similar to co-cultures containing enriched Mo (Table 2). Of note, by day 6, VEGF secretion in co-cultures with T cell-depleted PBMC was even higher in comparison to co-cultures containing enriched Mo (Table 2 and Supplementary Figure S2A). Although the difference was not significant, this suggests that in addition to Mo, other peripheral blood-derived cells may promote the production of VEGF when co-cultured with MSC. However, no vascular structures were detected in co-cultures of MSC with T cell-depleted PBMC (data not shown), even though these co-cultures generated extremely high VEGF levels. VEGF secretion by enriched Mo cultured for 24 h in 3D fibrin gels without MSC support was around 10-fold lower compared to co-cultures with MSC, and after 6 days of culture VEGF release by Mo was below detection level (Supplementary Figure S2C).

### CD31^bright^CD45^–^ Progenitor-Type Cells and T Cells Form Cell-in-Cell Structures in the Presence of MSC Within 3D Fibrin Gels

**FIGURE 5:**
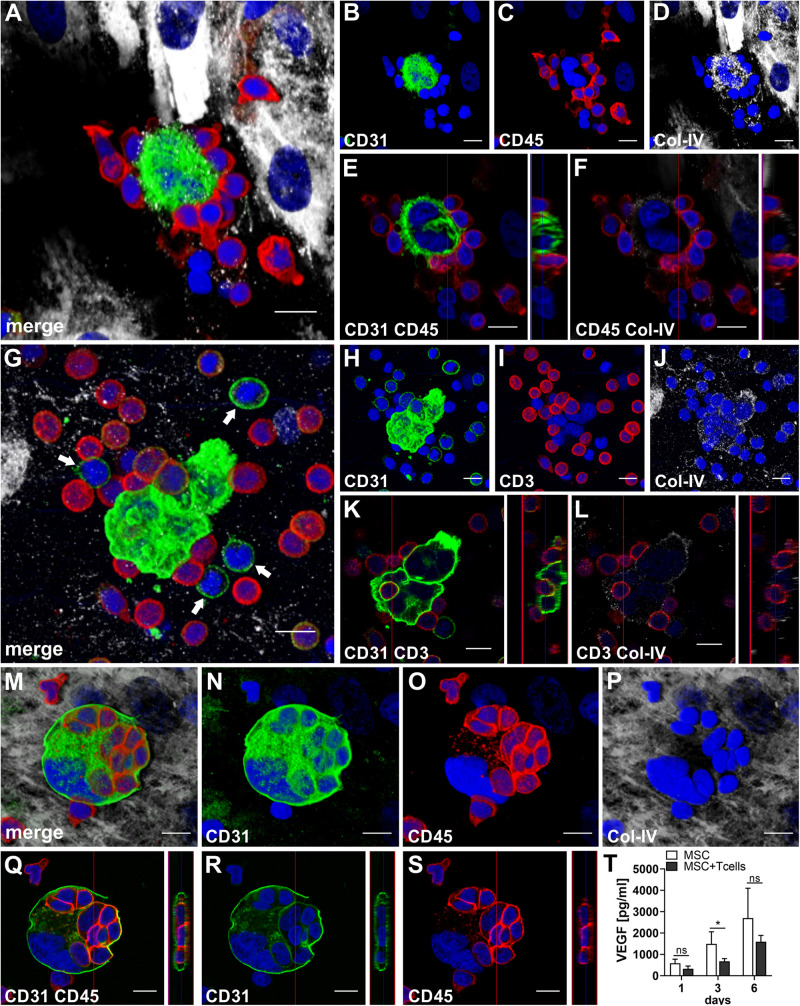
CD31^bright^CD45^-^ cells and T cells form cell-in-cell structures in 3D fibrin gels. Representative images from three separate co-culture experiments of MSC with enriched T cells are shown. **(A–F)** Experiment 1. CLSM images of intact fibrin gel on day 14 showing a bi-nucleated cell with **(B)** strong expression of CD31, surrounded by **(C)** CD45^+^ cells. The bi-nucleated cell does not express CD45, and is associated with **(D)** a fine Col-IV^+^ granular network. MSC also express Col-IV, but are CD31^-^. **(A)** Merge. **(A–D)** Nuclei stained with DAPI. Collapsed 11.9 μm-z-stack consisting of 17 consecutive images. Scale bars, 10 μm. **(E,F)** Orthoview CLSM images of collapsed z-stack shown in **(A–D)** demonstrating that **(E)** CD45^+^ cells are closely attached to the CD31^bright^ cell, and showing **(F)** CD45^+^ leukocytes associated with delicate granular Col-IV deposits surrounding the bi-nucleated cell. Scale bars, 10 μm. **(G–L)** Experiment 2. CLSM images of intact fibrin gel on day 6 showing two multi-nucleated cells **(H)** strongly expressing CD31, surrounded by **(I)** CD3^+^ T cells. Some CD3^+^ cells co-express CD31. The multi-nucleated cells are associated with a **(J)** Col-IV^+^ granular network. **(G)** Merge. Note the CD31^+^CD3^-^ mononuclear cells (arrows) in the surrounding of the multinucleated CD31^bright^ cells. **(G–J)** Nuclei stained with DAPI. Collapsed 17.28 μm-z-stack consisting of 36 consecutive images. Scale bars, 10 μm. **(K,L)** Orthoview CLSM images of collapsed z-stack shown in **(G–J)** demonstrating how **(K)** a CD3^+^ T cell is “pushing into” a multinucleated CD31^+^ cell, and showing **(L)** the fine Col-IV network around the two multi-nucleated cells. Scale bars, 10 μm. **(M–S)** Experiment 3. CLSM images of intact fibrin gel on day 14 showing a multi-nuclear cell with a diameter of 38 μm confined by a **(N)** CD31^+^ membrane and containing **(O)** several CD45^+^ cells. The multi-nuclear cell/structure is surrounded by **(P)** Col-IV^+^ MSC and matrix. **(M)** Merge. **(M–P)** Nuclei stained with DAPI. Collapsed 9.9 μm-z-stack consisting of 22 consecutive images. Scale bars, 10 μm. **(Q–S)** Orthoview CLSM images of collapsed z-stack shown in **(M–P)** demonstrating that **(Q)** CD45^+^ cells are internalized in the CD31^+^ cell building a cell-in-cell structure. **(R)** Internalized cells do not express CD31, and **(S)** the membrane of multicellular CD31^+^ structure is CD45^-^. **(Q–S)** Nuclei stained with DAPI. Scale bars, 10 μm. **(T)** Determination of VEGF by ELISA in cell-free supernatants of 3D MSC monocultures (*n* = 4) and corresponding co-cultures of MSC with enriched T cells, on day 1, 3, and 6, respectively. The data are expressed as mean values ± SD. *p value ≤ 0.05, ns = not significant.

To assess the contributions of T cells to neo-vessel formation and paracrine signature in the *in vitro* vascular niche environment, T cells were enriched by negative selection. Flow cytometry analyses showed that 96.4 ± 3.6% of cells expressed CD3 (Table 1). The enriched T cell fraction completely lacked CD14^+^ Mo and contained 0.40 ± 0.36% cells with the CD34^+^CD31^+^CD45^dim^CD14^–^ progenitor phenotype (Table 1). CLSM analyses of whole fibrin gels containing MSC in co-culture with enriched T cells for one to 2 weeks demonstrated a small number of prominent CD31^bright^CD45^–^ cells within the 3D matrix that measured >10 μm and partly contained two or more nuclei (Figure 5). Some of these CD31^+^ cells showed low expression of CD45 (Supplementary Animated z-stack S2, S3). CD31^bright^CD45^*dim*⁣/^^–^ cells were associated with a fine Col-IV^+^ network, and they were surrounded by CD31^–^Col-IV^+^ MSC and CD45^+^ leukocytes (Figures 5A–F and Supplementary Animated z-stacks S2, S3). CD45^+^ cells, identified as T cells by positive CD3 staining, seemed to “push into” the CD31^bright^ cells (Figures 5G–L and Supplementary Animated z-stacks S4, S5), and they were also found engulfed and internalized by CD31^bright^CD45^–^ cells thereby forming peculiar cell-in-cell structures (Figures 5M–P and Supplementary Animated z-stacks S6, S7). It appeared that CD45^+^ leukocytes/T cells were localized inside of vacuoles displaying a CD31^+^CD45^–^ membrane (Figures 5Q–S). Interestingly, cell-in-cell structures demonstrating CD3^+^ T cells engulfed by CD31^bright^ cells were also found in an autologous setting, when whole BM mononuclear cells were cultured for 2 weeks on fibronectin-coated slides (Supplementary Figure S3). As in co-cultures of MSC with enriched Mo, no vascular structures were detected in co-cultures of MSC with enriched T cells. The presence of T cells significantly decreased VEGF secretion by MSC at day 3 (Figure 5T). VEGF levels in co-cultures after 24 h and at day 6, respectively, were also lower in comparison to corresponding MSC mono-cultures, however, without reaching significance at these time points (Figure 5T and Table 2). The cytokine secretion pattern generated within the 3D fibrin environment by co-cultures of enriched T cells and MSC was similar to MSC mono-cultures, with the exception of an increase in CXCL1 and IL-8 levels and a slight decrease in CCL17 levels in MSC-T cell co-cultures (Figure 2). In 3D cultures of enriched T cells without MSC support, VEGF levels were below detection level at any time point and no cell-in-cell structures developed (data not shown).

### Complex Vascular Structures Develop in 3D Fibrin Matrices When MSC Are Co-cultured With Both Enriched Mo and T Cells

**FIGURE 6:**
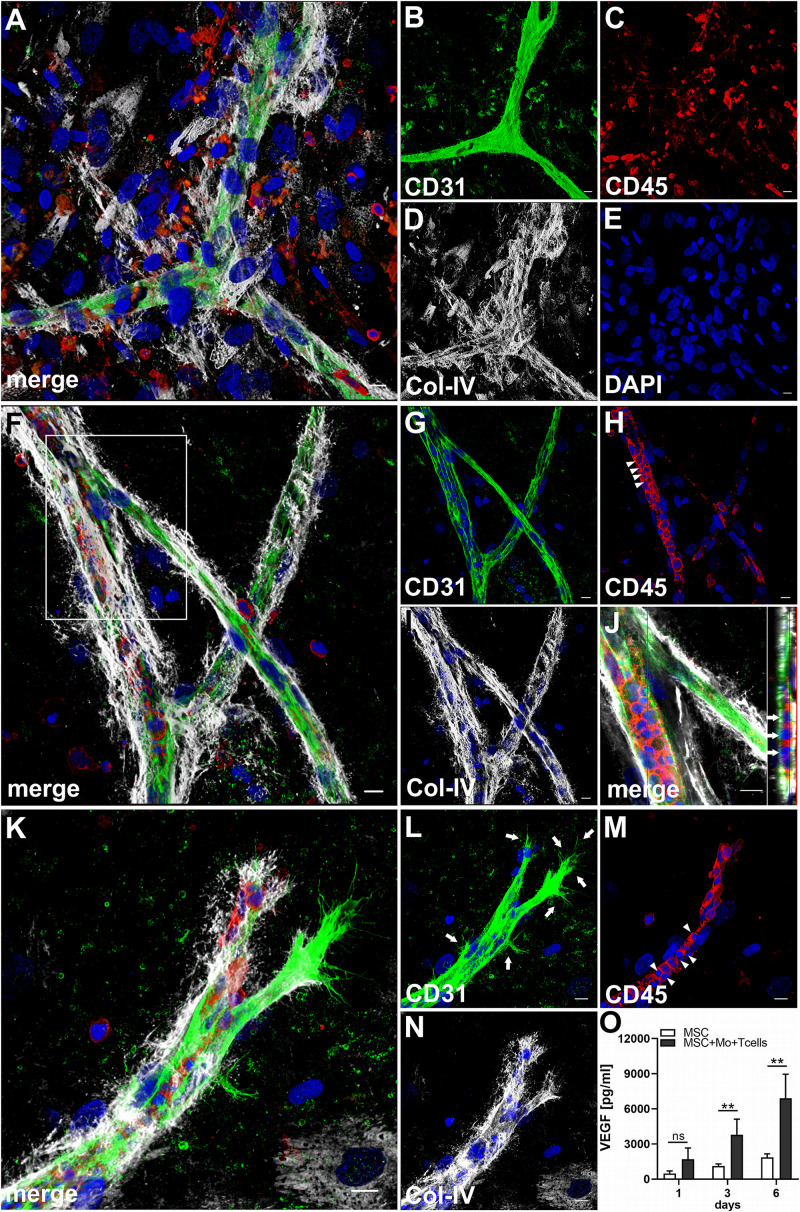
Co-culture of MSC, enriched Mo and enriched T cells leads to the development of complex vascular structures in 3D fibrin gels. Representative images from two separate co-culture experiments of MSC with both enriched Mo and enriched T cells. **(A–E)** Experiment 1. CLSM images of intact fibrin gel on day 14 showing a **(B)** CD31^+^ branched vascular structure surrounded by **(D)** Col-IV^+^ matrix and cells. CD31^+^ EC also co-express Col-IV. **(C)** CD45^+^ leukocytes in the vicinity of the neo-vessel co-express intermediate levels of CD31. **(A)** Merge. **(E)** DAPI nuclear stain. **(A–E)** Collapsed 20.25 μm-z-stack consisting of 45 consecutive images. Scale bars, 10 μm. **(F–N)** Experiment 2. CLSM images of intact fibrin gel on day 14 demonstrating that **(G)** the CD31^+^ vascular tubes show two branch points, contain **(H)** several CD45^+^ leukocytes, some of which show condensed nuclei (arrowheads), and is surrounded by **(I)** Col-IV^+^ matrix and cells. **(J)** Orthoview CLSM image of collapsed z-stack shown in [**(F)** boxed area] confirming that CD45^+^ leukocytes are localized inside of the developing neo-vessel (arrows). **(F)** Merge. **(F–J)** DAPI nuclear stain. **(F–I)** Collapsed 16 μm-z-stack consisting of 40 consecutive images. Scale bars, 10 μm. **(K–N)** Leading edge of a neo-vessel demonstrating **(L)** two CD31^+^ EC at the tip of the vascular structure showing numerous filopodial protrusions (arrows). Proximal EC show fewer filopodia (arrows). **(M)** CD45^+^ leukocytes are closely associated with one of the “tip cells” of the growing neo-vessel, and they are present inside of the vascular structure, where some of them show condensed nuclei (arrowheads). The vascular tube is surrounded by **(N)** Col-IV^+^ matrix building a fine Col-IV^+^ network especially toward one of the tip cells of the neo-vessel. **(K)** Merge. **(K–N)** DAPI nuclear stain. Collapsed 9.75 μm-z-stack consisting of 25 consecutive images. Scale bars, 10 μm. **(O)** Determination of VEGF by ELISA in cell-free supernatants of 3D MSC monocultures (*n* = 4) and corresponding co-cultures of MSC with both enriched Mo and enriched T cells, on day 1, 3, and 6, respectively. The data are expressed as mean values ± SD. **p value ≤ 0.01, ns = not significant.

When MSC were co-cultured with enriched Mo and enriched T cells, complex vessel structures developed in the 3D fibrin gels within 1–2 weeks (Figure 6 and Supplementary Animated z-stack S8), suggesting that synergistic interaction effects between T cells, Mo, progenitor cells and MSC were required for neo-vessel formation. Neo-vessels showed several branch points, consisted of CD31^+^ EC (Figure 6B) that co-expressed Col-IV (Figure 6D), and were surrounded by CD31^–^Col-IV^+^ MSC and Col-IV^+^ matrix (Figure 6D and Supplementary Figure S4B,D). The vascular tubes contained CD45^+^CD31^–^ hematopoietic cells (Figure 6H) localized inside of CD31-lined intracellular spaces adjacent to CD31^+^CD45^–^ EC (Figures 6F,J and Supplementary Animated z-stack S9). Some of these “intra-vascular” CD45^+^ cells showed condensed nuclei (Figure 6H) indicating that they underwent apoptosis. EC of newly forming vessels, and in particular, CD31^+^ cells at the leading edge of neo-vessels showed CD31^+^ filopodial protrusions (Figures 6K,L) that resembled tip cells during angiogenic sprouting. Filopodial protrusions were surrounded by a fine Col-IV^+^ network (Figure 6N and Supplementary Animated z-stack S10), and CD45^+^ leukocytes were found in their vicinity (Figure 6M). In the surrounding of neo-vascular structures, numerous irregular-shaped CD31^+^CD45^+^ cells resembling macrophages were found in close vicinity to Col-IV^+^ MSC (Figures 6A–E and Supplementary Animated z-stack S8). This cellular distribution pattern was reminiscent of a pattern found in 3D synovial explant cultures (Rüger et al., 2018), where vascular outgrowth was also associated with surrounding stromal cells in close contact with CD31^+^CD45^+^ cells (Supplementary Figure S4E–I and Supplementary Animated z-stack S11). VEGF secretion progressively increased during communication and co-operation of MSC with enriched Mo and T cells, showing significantly higher levels than corresponding cultures of MSC without Mo and T cells from day 3 onward (Figure 6O and Table 2). VEGF release in co-cultures of Mo and T cells without support of MSC measured on day 6 was below detection level (data not shown). The cytokine secretion profile resulting from interaction of MSC with enriched T cells together with enriched Mo for 24 h showed strong similarities to the secretion profile of MSC co-cultured with enriched Mo (Figure 2). However, while CXCL10 - an anti-angiogenic cytokine - was detected at high levels in co-cultures of MSC with enriched Mo, the concomitant presence of enriched T cells in the co-cultures greatly diminished CXCL10 secretion (Figure 2).

### CD14^+^ Monocytes Are Not Required for Vascular Morphogenesis

**FIGURE 7:**
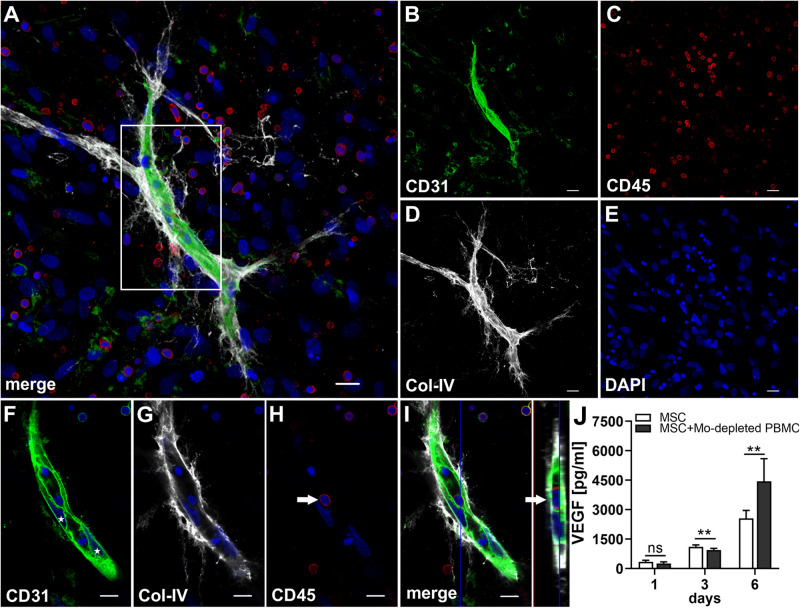
Vascular tubes develop in the absence of CD14^+^ Mo. Representative CLSM images of MSC co-cultured with Mo-depleted PBMC in 3D fibrin matrix on day 14 showing **(B)** a CD31^+^ vascular structure amongst **(C)** numerous CD45^+^ leukocytes. **(D)** Col-IV matrix staining alongside the developing vessel extends beyond the CD31^+^ vascular structure. **(A)** Merge. **(E)** DAPI nuclear stain. **(A–E)** Collapsed 13.5 μm-z-stack consisting of 27 consecutive images. Scale bars, 20 μm. **(F–H)** 2D cross-section images (frame 10/27) of the boxed area in panel **(A)** showing the core of the vascular structure with **(F)** parietally located CD31^+^ EC (stars), tightly connected and aligned with **(G)** Col-IV matrix, and associated with **(H)** CD45^+^ leukocytes. **(I)** Orthoview CLSM image of collapsed z-stack shown in [**(A)**, boxed area] demonstrating that the CD45^+^ leukocyte [arrow in panel **(H)**] is present inside of a CD31-lined vacuole adjacent to a CD31^+^CD45^-^ EC (arrow). **(F–I)** Nuclei stained with DAPI. Scale bars, 10 μm. **(J)** Determination of VEGF by ELISA in cell-free supernatants of 3D MSC monocultures (*n* = 6) and corresponding co-cultures of MSC with Mo-depleted PBMC, on day 1, 3, and 6, respectively. The data are expressed as mean values ± SD. **p value ≤ 0.01, ns = not significant.

In order to investigate whether Mo are required for the physical assembly of neo-vessels, PBMC were depleted from Mo using a CD36 antibody prior to co-culture with MSC. Flow cytometry analyses revealed that Mo-depleted PBMC contained 0.32 ± 0.20% cells with the progenitor phenotype CD34^+^CD31^+^CD45^dim^CD14^–^ and 69.0 ± 12.2% CD3^+^ cells (Table 1). Despite the absence of CD14^+^ Mo, Mo-depleted PBMC in co-culture with MSC generated CD31^+^ vascular structures surrounded by Col-IV^+^ matrix in close vicinity to CD45^+^ leukocytes (Figure 7). CD45^+^ leukocytes were also found inside of developing vascular tubes within CD31-lined intracellular vacuoles (Figure 7I, Supplementary Figure S5 and Supplementary Animated z-stacks S12, S13). The morphology of the vascular structures strongly resembled the features of neo-vessels developing in co-cultures of MSC with PBMC and enriched Mo together with enriched T cells, respectively, suggesting that classical Mo do not play a direct role in the assembly of vascular structures. Vascular tubes were found in close contact and perfectly aligned with Col-IV^+^ matrix, and collapsed z-stack images demonstrated that Col-IV^+^ matrix structures extended beyond the CD31^+^ vascular structure suggesting that MSC-mediated mechanical signals and guiding cues may promote tube elongation in the 3D fibrin environment (Figures 7A,B,D and Supplementary Animated z-stack S12). VEGF release in co-cultures of MSC with Mo-depleted PBMC was significantly decreased at day 3, when compared to corresponding MSC monocultures (Figure 7J and Table 2), showing similarities to co-cultures of MSC with enriched T cells (Figure 5T). However, while the presence of T cells decreased VEGF secretion by MSC at all time points measured, co-cultures of MSC with Mo-depleted PBMC generated significantly higher levels of VEGF by day 6 compared to corresponding MSC monocultures (Figure 7J). Furthermore, co-cultures of MSC with Mo-depleted PBMC released significantly higher amounts of VEGF in comparison to MSC-T cell co-cultures from day 3 onward (Supplementary Figure S2B) suggesting that other non-myeloid cells contributed to VEGF release in these co-cultures thereby promoting the formation of vascular structures (Table 2).

### Lumens Appear to Form Through Coalescence of Intracellular Vacuoles and Apoptosis of Leukocytes

**FIGURE 8:**
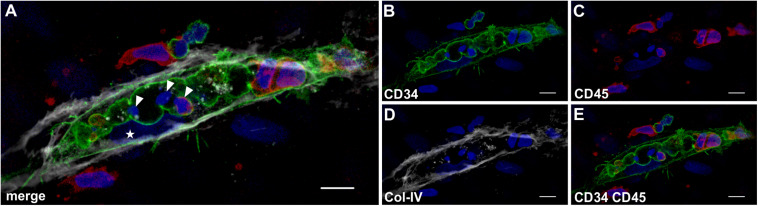
Lumen formation occurs through coalescence of intracellular vacuoles and apoptosis of leukocytes. Representative CLSM images of MSC co-cultured with PBMC in 3D fibrin matrix showing **(A)** a multi-cellular structure confined by a **(B)** CD34^+^ membrane separating a centrally located space apparently created by coalescing vacuoles that contains **(C)** CD45^+^ leukocytes. The CD34^+^ cell [star in panel **(A)**] is part of the parietal EC lining that is closely associated with **(D)** the adjacent Col-IV^+^ matrix. **(E)** CD34^+^ cells do not co-express CD45. **(A)** The merged image demonstrates three cells within the coalescing vacuoles showing condensed nuclei (arrowheads) consistent with apoptosis. Note the elongated cell between vacuole and Col-IV matrix (star) resembling an EC attached to the basement membrane. Scale bars, 10 μm.

In all co-cultures that generated vascular structures, the developing endothelial cords displayed similar characteristic features. They showed large coalescing intracellular vacuoles lined with a membrane expressing CD31 (Figure 7F) and CD34 (Figure 8B) associated with abluminal Col-IV deposition (Figure 7G) as well as intra-vacuolar granular Col-IV deposits (Figure 8D). Vacuoles contained CD45^+^ leukocytes, with adjacent parietally located CD31^+^/CD34^+^ EC (Figure 7, 8). A number of these “intravascular” cells displayed condensed nuclei implying they underwent apoptosis thereby conceivably contributing to the formation of a luminal space (Figure 8).

## Discussion

Tissue damage is commonly associated with an initial inflammatory reaction. During the repair process immune cells infiltrating the affected area come in contact with tissue-resident cells, and communicate with MSC and EPC co-recruited from the circulation. The perivascular deposition of a fibrin matrix creates the basic scaffold where new blood vessels can form and contribute to the healing process to re-establish homeostasis. In our study, we have established a fibrin-based xeno-free human vascular niche model in which we cultivated, in 3D, MSC with PBMC in order to investigate *de novo* vessel formation in an inflammatory environment closely mimicking the *in vivo* setting. We demonstrated that complex vascular structures can form in a 3D fibrin environment in the absence of pre-existing EC or vascular structures, and that this process is carried out exclusively through complex cell-cell communication and co-operation amongst MSC, mature PBMC and circulation-derived progenitor cells. Results from gender-mismatched co-cultures demonstrated that EC of vascular structures generated in the 3D fibrin matrices originated from PBMC, suggesting *in situ* differentiation of blood-derived progenitor cells within the fibrin niche environment.

Monocytes and T cells are major players during repair processes, therefore we investigated their specific contributions to neo-vessel formation in the vascular niche model. We observed that neo-vessels formed only in co-cultures of MSC and enriched Mo mixed with enriched T cells, implying a synergistic effect, where Mo and T cell interactions with blood-derived progenitor cells and MSC complemented each other to create conditions allowing for the development of vascular structures in the fibrin environment. Microenvironmental factors, such as the macromolecular concentration of the matrix, as well as cell number influence the phenotype of MSC and play a key role in promoting angiogenesis *in vivo*, as shown e.g., in a hindlimb ischemia model where formation of new blood vessels was accelerated when MSC were delivered at a low cell dose in soft matrix (Thomas et al., 2020). In order to mimic the situation *in vivo*, in our *in vitro* study we set up fibrin scaffolds using a fibrinogen concentration that resembles circulating fibrinogen levels in the blood (2–4 mg/ml) creating malleable and translucent gels, with 3D co-cultures containing 100 times more PBMC (5 × 10^6^) compared to the number of MSC (5 × 10^4^). No vascular tubes developed when MSC were co-cultured exclusively with enriched Mo, even though enriched Mo contained significantly higher numbers of progenitor cells compared to whole PBMC. The contribution of Mo to neo-vessel formation was essentially to create a pro-angiogenic paracrine niche environment via cross-talk with MSC reflected by the secretion of several mediators typically found during inflammatory neo-vessel formation *in vivo* (Szade et al., 2015) including high levels of VEGF (Figure 2 and Table 2). On the other hand, cross-talk of enriched T cells with blood-derived progenitor cells appeared to be crucial for the vascular morphogenesis process *per se*. Although co-cultures of MSC with enriched T cells alone did not generate vascular structures, a small number of conspicuously bright CD31^+^CD45^–^ cells, some showing weak CD45 expression, was found in the 3D matrix within 1 week of culture. The phenotype of these cells demonstrated that they were circulation-derived, possibly originating from CD31^+^CD34^+^CD45^dim^CD14^–^ progenitor cells present in peripheral blood. These CD31^+^CD45^–^ cells were closely associated with T cells and partially engulfed them, leading to the formation of cell-in-cell structures. Evidence for a co-operative mechanism involving blood-derived progenitor cells and differentiated immune cells was originally presented in a seminal publication by Asahara et al. (1997), where the concept of circulating EPC was first introduced. The authors showed that cord-like structures and network formation *in vitro* on fibronectin-coated plates occurred only when blood-derived CD34^+^ progenitor cells were co-cultured with CD34^–^ cells. Accordingly, we suggest that cross-talk of T cells with progenitor cells could have a functional relationship to differentiation of EPC toward mature EC, and that the process of T cell internalization by progenitor cells might be the initial cue for subsequent EC differentiation in a conducive pro-angiogenic paracrine environment provided by cross-talk of MSC with Mo. Several studies have shown that T cells are the main mature leukocyte subset present in early EPC colonies (Hur et al., 2007; Rohde et al., 2007), and the generation of ECFC requires the presence of T cells (Wilde et al., 2016). Yet, the origin of circulating progenitor cells giving rise to ECFC has not been fully clarified. Furthermore, their phenotype is still elusive and might not be the same as the phenotype of ECFC generated by culture of whole PBMC. In contrast to mature EC, ECFC are highly proliferative cells, however, they are phenotypically indistinguishable from mature EC and do not express hematopoietic markers including CD45. A recently published consensus statement concerning EPC criteria discriminates ECFC/’true EPC’ (CD31^+^CD146^+^CD45^–^) from myeloid angiogenic cells which are of hematopoietic origin (i.e., they express CD45 and CD14) (Medina et al., 2017). In our study, the CD31^+^CD34^+^CD45^dim^CD14^–^ progenitor cells present in LRS chambers showed an expression profile partially related to both endothelial and hematopoietic lineage cells. In addition, we have detected cells with this phenotype also in *ex vivo* samples of human BM (unpublished observation), and a recent study has demonstrated that BM-derived hematopoietic stem cells contribute to vascular network formation *in vitro* when co-cultured with BM-MSC (Sasse et al., 2019). It is tempting to speculate that CD31^+^CD34^+^CD45^dim^CD14^–^ progenitor cells may represent the circulating ECFC progenitors that differentiate *in situ* through cell-in-cell structure formation with T cells once they are recruited to a vascular niche environment where they lose CD45 expression. This line of thought is supported by the presence of CD31^+^CD45^+^ cells in human BM that can contribute to blood vessel formation by differentiation into mature EC (Kim et al., 2010), and may reconcile conflicting reports about the origin of ECFC in tissue vascular niches (Ingram et al., 2005; Duong et al., 2011; Alphonse et al., 2015) and in the BM (Lin et al., 2000). Moreover, ECFC can be manufactured from steady-state leukapheresis using a program intended for the collection of mononuclear cells including hematopoietic progenitor cells (Siegel et al., 2018), providing additional support for *in situ* development of ECFC from PB-derived progenitor cells that subsequently generate vascular structures in co-operation with MSC as demonstrated in our *in vitro* 3D fibrin model. Interestingly, in our study we could show that human BM harbors CD31^+^ cells that interact with and internalize T cells in culture on fibronectin-coated slides, demonstrating co-operation of progenitor cells and differentiated cells via cell-in-cell structure formation in an autologous setting (Supplementary Figure S3). Cell-in-cell structure formation defines a process by which one or more cells penetrate into the cytoplasm of another cell causing cell structure and biological alteration. Although this phenomenon has been revealed nearly 100 years ago under pathological circumstances (Lewis, 1925), only few reports about its biological significance have been published thus far. Yet, cell-in-cell structures definitely represent a specific characteristic feature of several pathological conditions, including Rosai-Dorfman disease, chronic myeloproliferative diseases and some other hematological diseases (Shamoto, 1981; Thiele et al., 1984; Deshpande et al., 2000; Schmitt et al., 2002). In addition, cell-in-cell structure formation occurs during murine T cell development in the thymus, where thymocyte nurse cells internalize immature T cells within cytoplasmatic vacuoles to nurture and educate them into mature T cells (Wekerle et al., 1980), clearly pointing to the specific physiological role of cell-in-cell structures. Similar may also apply to our vascular niche model, where T cell internalization within vacuoles of progenitor-type cells appears to initiate neo-vessel development.

Apart from their role in stabilizing newly formed blood vessels, MSC are known to produce a number of paracrine factors that support new vessel formation (Caplan and Correa, 2011; Watt et al., 2013). Here, we could show that within 24 h, MSC in 3D fibrin matrices produce a considerable number of chemokines, cytokines and growth factors, and several mediators related to inflammatory neo-vessel formation are upregulated when matrix-embedded MSC come in contact with leukocytes, in agreement with previous findings (Szekanecz and Koch, 2001). Amongst the pro-angiogenic mediators, VEGF is one of the most potent inducers of vascular growth, whereby e.g., disruption of a single VEGF allele in mice is embryonically lethal (Carmeliet et al., 1996; Ferrara et al., 1996). In our study, cross-talk of MSC with either Mo or T cells influenced the paracrine secretion pattern including VEGF levels in the niche in distinctive ways. Cross-talk of MSC with Mo increased VEGF secretion in the niche (Figure 4F), and as shown by transwell experiment, increased VEGF release was predominantly due to physical interaction (Figure 4G). On the other hand, cross-talk of MSC with T cells had the opposite effect and cells in the 3D niche secreted lower VEGF levels compared to corresponding MSC mono-cultures (Figure 5T). When PBMC were depleted from Mo, VEGF release after 24 h was similar to co-cultures of MSC with enriched T cells (Table 2). However, from day 3 onward, VEGF levels were significantly higher (Supplementary Figure S2B), suggesting that immune cells, other than Mo, compensated for decreased VEGF levels caused by T cell cross-talk with MSC. In contrast, depletion of T cells from PBMC increased the percentage of Mo (Table 1), and co-culture of T cell-depleted PBMC with MSC resulted in even higher VEGF levels on day 6 in comparison with co-cultures containing enriched Mo (Table 2 and Supplementary Figure S2A), consistent with the notion that immune cells other than Mo also contribute to VEGF secretion via cross-talk with MSC. Accordingly, co-cultures of MSC with whole PBMC and T cell-depleted PBMC generated the highest VEGF levels (Table 2); however, no vascular structures were formed in co-cultures lacking T cells. In comparison, cross-talk of MSC with Mo-depleted PBMC led to the development of neo-vessels despite lower VEGF levels (Figure 7), demonstrating that classical Mo are not mandatory for the formation of neo-vascular structures *per se*, and confirming also the important role of T cells in vascular morphogenesis. These findings further make evident that successful neo-vessel formation requires both a pro-angiogenic paracrine niche environment, e.g., sufficient VEGF levels, as well as key cellular players, e.g., T cells. We have previously shown that VEGF release by BM-derived MSC cultured in 3D fibrin matrices increases significantly under stimulation with inflammatory cytokines, such as TNF-α and IFN-γ, but no vascular structures form in the absence of PBMC (Rüger et al., 2018). This previous study was conducted using medium supplemented with fetal bovine serum, and MSC and PBMC in co-culture formed vasculogenic clusters and cells with endothelial phenotype emerging from them (Rüger et al., 2018). These vascular structures, however, did not reach the complexity of neo-vessels generated under xeno-free conditions as demonstrated in this study.

The assembly of a basement membrane is an essential step in the maturation of new blood vessels as it mediates tissue compartmentalization. The vascular basement membrane interacts directly with the pericytes/MSC on the outside, and the EC that line the inside of the blood vessel. Col-IV, a major constituent of basement membranes, plays a key role in blood vessel morphogenesis and is essential for blood vessel stability (Poschl et al., 2004). Apart from their role as support matrix, recent *in vivo* studies have shown that basement membranes actively shape tissue morphology (Morrissey and Sherwood, 2015). Here we observed association of Col-IV matrix with cell-in-cell structures (Figure 5 and Supplementary Animated z-stack S7), as well as with EC and MSC surrounding the developing vascular structures (Supplementary Animated z-stacks S1, S8, S9, S12, S13), supporting the concept that both cellular compartments, i.e., EC and MSC contribute to the assembly of a basement membrane necessary for EC tube formation and vascular development (Stratman and Davis, 2012). Previous work has reported that ECFC deposit matrix proteins including Col-IV organized in a web-like structure (Kusuma et al., 2012), and we have shown recently (Rüger et al., 2018) and also in this study that MSC produce and deposit Col-IV when cultured in fibrin gels. In addition to their contribution to establish a basement membrane, mechanical tension exerted to the 3D matrix by MSC may provide signals promoting elongation of the cell-in-cell structures and thereby physically guide and mediate the formation of vascular tubes, a view consistent with the concept of biomechanical forces regulating tissue vascularization (Ingber, 2002; Kilarski et al., 2009; Mammoto et al., 2009). In this context, CD31^+^ filopodial protrusions formed by neo-vessel EC (Figure 6L and Supplementary Animated z-stack S10) may act as points of attachment to the Col-IV matrix, in order to extend the vascular tube and stabilize it as it moves (DeLisser, 2011).

Lumen formation is essential for the generation of a functional vascular system, and in both vasculogenesis and angiogenesis, it takes place in a cord of EC. Different mechanisms of lumen formation have been proposed including cord hollowing and cell hollowing (Iruela-Arispe and Davis, 2009). The current study provides evidence that coalescence of vacuoles generated by CD31^bright^ progenitor cells through engulfment of T cells can contribute to lumen formation in association with apoptosis of engulfed cells (Figure 8). The results are consistent with the vacuole coalescence model, where EC form large intracellular vacuoles, which constitute a central vascular lumen inside each EC, thus giving rise to a seamless vascular lumen (Folkman and Haudenschild, 1980; Davis and Camarillo, 1996; Kamei et al., 2006). Two of our previous studies have demonstrated the presence of hematopoietic cells within developing vascular structures. We have shown that BM-derived mononuclear cells can form vascular structures through self-organization in 3D fibrin gels, originating from cell clusters, and that some of these neo-vessels contain CD45^+^ hematopoietic cells (Ruger et al., 2008). Using a tissue explant model, we have recently demonstrated that vascular outgrowth from synovial tissue samples embedded in fibrin matrices is dependent on the presence of inflammatory cells, and that developing vascular sprouts contain intraluminal CD45^+^ cells together with apoptotic cells (Rüger et al., 2018), supporting the notion that lumen formation is associated with apoptosis.

Cell-cell interaction and self-organization within physiologically relevant 3D microenvironments have been the focus of a number of recently published co-culture models for vascular tissue engineering. Co-culture of microvascular EC and MSC, both derived from adipose tissue, has been shown to generate complex vascular networks within biodegradable 3D poly-L-lactic acid (PLLA)/poly-lactic-co-glycolic acid (PLGA) constructs (Freiman et al., 2016). Interestingly, we observed that adipose-derived MSC co-cultured with PBMC in 3D fibrin matrices generate neo-vascular structures similar to neo-vessels formed by PB-derived progenitor cells in the presence of BM-derived MSC as shown in this study (unpublished observation). While our 3D model has the limitation of being a static system, microfluidic technology represents a promising platform providing insight into the spatio/temporal dynamics of vascular cell behavior. Using a microfluidic system, Yamamoto et al. have shown that co-culture of human umbilical vein endothelial cells (HUVEC) and human BM-derived MSC forms stable, branching capillary networks in collagen matrices, covered by MSC-derived pericytes and with continuous lumens of <10 μm diameter, comparable to the size of capillaries *in vivo* (Yamamoto et al., 2018). In an innovative blood-brain barrier microvascular network model developed to mimic aspects of the organization and structure of the brain microcirculation observed *in vivo*, stable and perfusable microvascular networks formed through self-assembly of human induced pluripotent stem cell-derived endothelial cells (iPSC-EC), brain pericytes and astrocytes co-cultured together in a 3D fibrin matrix (Campisi et al., 2018). Vascular networks in both studies, generated in microfluidic devices, showed morphological similarities to the neo-vessel structures generated in our 3D model, including branching, formation of lumens of <10 μm diameter and deposition of basement membrane proteins, such as Col-IV. A major difference between our model and the two models using microfluidic devices, is - apart from the methodology - the composition of cell types used for co-culture with MSC. While HUVEC and iPSC-EC, respectively, were included in the two microfluidic models, the initial co-culture set-up in our model did not include EC. The aim of our study was to investigate mechanisms of vasculogenesis by *ex vivo* PB-derived progenitor cells in an inflammatory context, including specific contributions of Mo and T cells in this process. For this reason, we co-cultured MSC with PB-derived cells obtained from LRS chambers that contain increased numbers of viable hematopoietic CD34^+^ progenitor cells (Néron et al., 2007).

## Conclusion

In conclusion, in our study, using the xeno-free 3D vascular niche model, we have applied a bottom-up approach that relied on encouraging cells to recapitulate physiological mechanisms of neo-vessel formation, occurring both during development and tissue repair, i.e., *de novo* vessel formation through vasculogenesis. We purposely chose not to include mature EC or culture-derived ECFC in the culture set up. This allowed us, to investigate the potential of peripheral blood-derived progenitor cells obtained *ex vivo* from the circulation, to differentiate into mature EC, when cultured together with mature circulating immune cells and MSC in a 3D fibrin environment. Here, we have demonstrated that vascular structures can form *de novo* through interaction and co-operation of blood-derived progenitor cells with mature immune cells from the circulation, in the presence of MSC, and in the context of a permissive niche environment (3D fibrin matrix). More specifically, the data might indicate that blood-derived progenitors recruited to perivascular niches can change their phenotype, and upon interaction with T cells and supportive MSC, develop into vascular EC *in situ*. Although predominantly descriptive, the niche model used in our study reveals that a complex synergistic network of cellular, extracellular and paracrine cross-talk can contribute to *de novo* vascular development through self-organization. Reproduction in animal models are planned in order to convincingly show that *de novo* formed vascular structures as presented in this *in vitro* model can integrate into a pre-existing vascular system *in vivo*. Altogether, our findings demonstrate an innovative *in vitro* setup enabling *de novo* vessel formation through vasculogenesis, that would be expected to have *in vivo* relevance and that may open up an additional perspective for the successful engineering of autologous blood vessels for therapeutic purposes.

## Data Availability Statement

The raw data supporting the conclusions of this article will be made available by the authors, without undue reservation.

## Ethics Statement

The studies involving human participants were reviewed and approved by Ethics Committee of the Medical University of Vienna. The patients/participants provided their written informed consent to participate in this study.

## Author Contributions

BR conceived the work, designed the study, generated, analyzed, and interpreted the data, and wrote the manuscript. JB and MF contributed to the design of the study. TB generated, analyzed, and interpreted the data. E-MD performed the genetic analyses. MP created the heat map and analyzed the flow cytometry data. TB, PU, MF, JB, and GL revised the work for intellectual content. All authors contributed to the manuscript, read, and approved the submitted version.

## Conflict of Interest

The authors declare that the research was conducted in the absence of any commercial or financial relationships that could be construed as a potential conflict of interest.
