# Supplementary material for: De novo Vessel Formation Through Cross-Talk of Blood-Derived Cells and Mesenchymal Stromal Cells in the Absence of Pre-existing Vascular Structures
Source: Front Bioeng Biotechnol. 2020 Nov 16;8:602210. doi: 10.3389/fbioe.2020.602210 (PMC7718010; doi:10.3389/fbioe.2020.602210)
Supplement: Supplementary file 1 [file Data_Sheet_1.zip › Supplemental Figure 1.pdf]

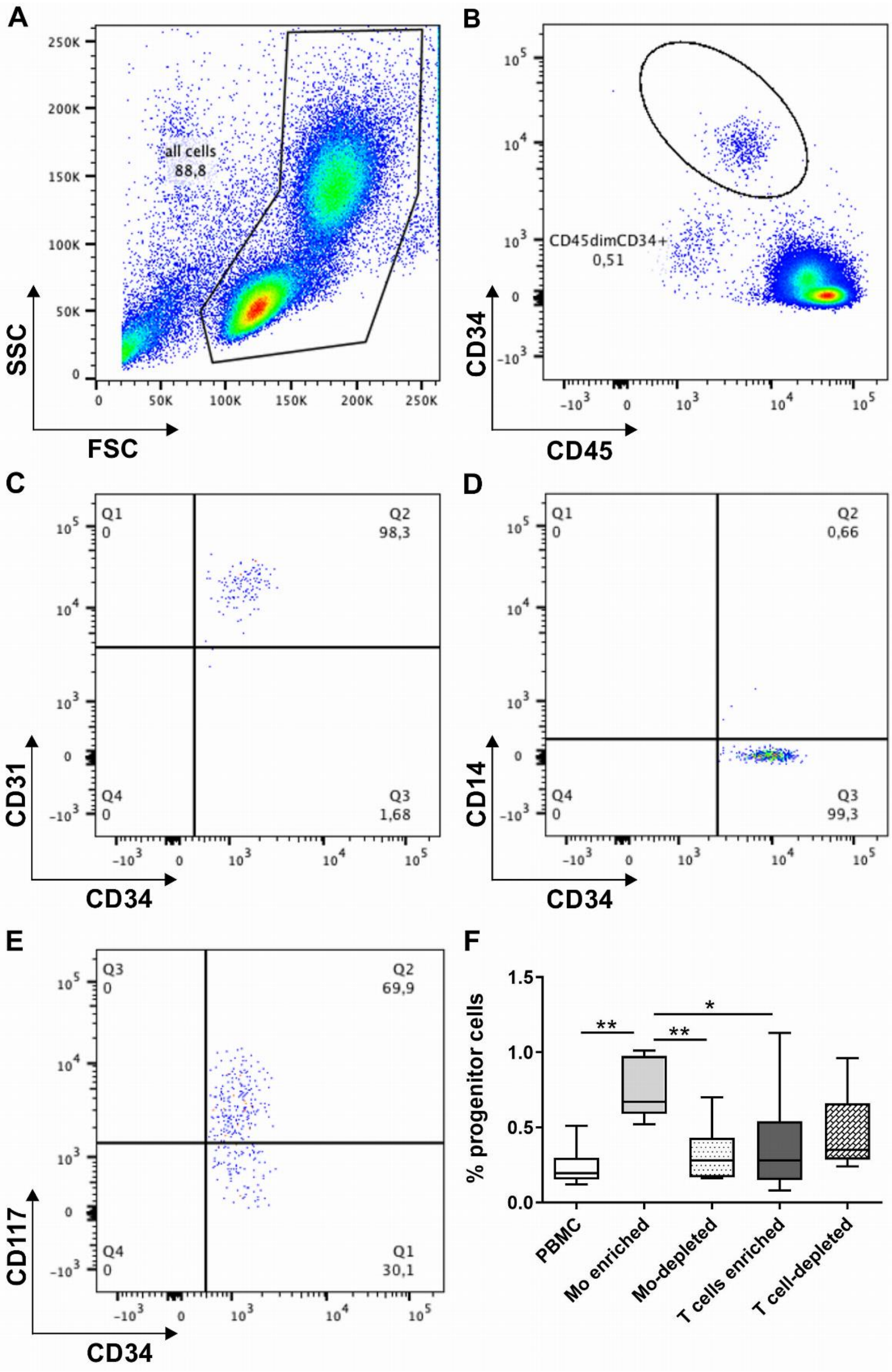

Supplemental Figure 1. Peripheral blood obtained from LRS chambers contains mononuclear cells with progenitor phenotype. Flow cytometry analyses of PBMC isolated from LRS chambers. **(A)** Forward/Side Scatter. **(B)** CD34$^+$ cells show low-level expression of CD45. **(C)** CD34$^+$ cells co-express CD31. **(D)** CD34$^+$ cells do not express CD14. **(E)** Co-expression of CD117 by the majority of CD34$^+$ cells. **(F)** Percentage of CD34$^+$CD31$^+$CD45^{dim}CD14$^-$ progenitor-type cells in immune cell fractions. The data are expressed as mean values±SD. *p value ≤0.05, ** p value ≤0.01.
