# Supplementary material for: De novo Vessel Formation Through Cross-Talk of Blood-Derived Cells and Mesenchymal Stromal Cells in the Absence of Pre-existing Vascular Structures
Source: Front Bioeng Biotechnol. 2020 Nov 16;8:602210. doi: 10.3389/fbioe.2020.602210 (PMC7718010; doi:10.3389/fbioe.2020.602210)
Supplement: Supplementary file 1 [file Data_Sheet_1.zip › Supplemental Figure 2.PDF]

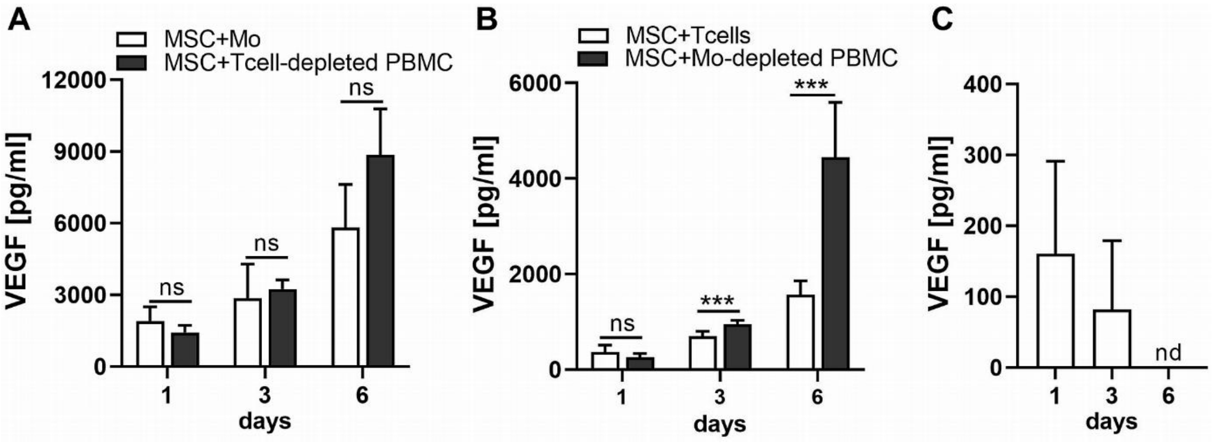

Supplemental Figure 2. (A) Determination of VEGF by ELISA in cell-free supernatants of MSC co-cultured with enriched Mo (n=4) and MSC co-cultured with T cell-depleted PBMC (n=4) in 3D fibrin matrix on day 1, day 3 and day 6, respectively. The data are expressed as mean values±SD. ns=not significant. **(B)** Determination of VEGF by ELISA in cell-free supernatants of MSC co-cultured with enriched T cells (n=6) and MSC co-cultured with Mo-depleted PBMC (n=6) in 3D fibrin matrix on day 1, day 3 and day 6, respectively. The data are expressed as mean values±SD. ns=not significant, ***p value ≤0.001. **(C)** Determination of VEGF by ELISA in cell-free supernatants of enriched Mo (n=3) without stromal support in 3D fibrin matrix on day 1, day 3 and day 6, respectively. nd=not detected.
