# Supplementary material for: De novo Vessel Formation Through Cross-Talk of Blood-Derived Cells and Mesenchymal Stromal Cells in the Absence of Pre-existing Vascular Structures
Source: Front Bioeng Biotechnol. 2020 Nov 16;8:602210. doi: 10.3389/fbioe.2020.602210 (PMC7718010; doi:10.3389/fbioe.2020.602210)
Supplement: Supplementary file 1 [file Data_Sheet_1.zip › Supplemental Figure 3.PDF]

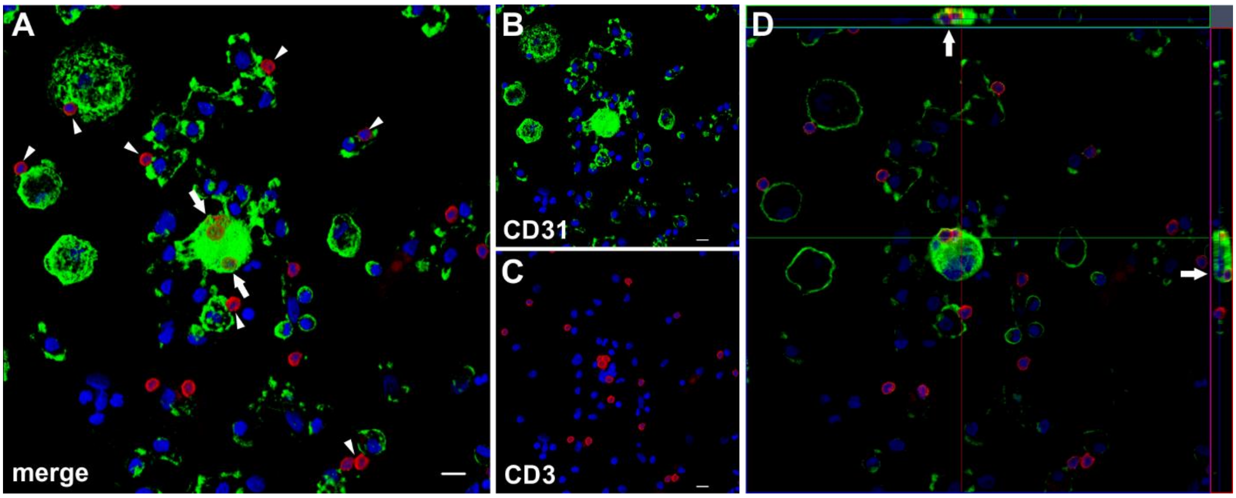

Supplemental Figure 3. Formation of cell-in-cell structures during culture of whole BM mononuclear cells on fibronectin-coated slides. Whole BM mononuclear cells were cultured for two weeks on fibronectin-coated chamber slides (Thermo Fisher Scientific Nunc Lab-Tek Chamber Slide System, Waltham, MA, USA) using complete αMEM medium supplemented with 10% human AB serum without externally added growth factors. Immunofluorescence staining of paraformaldehyde-fixed cells demonstrating **(B)** numerous $CD31^{bright}$ cells closely interacting with **(C)** $CD3^{+}$ T cells. **(A)** Merge. Note the close contact of T cells with $CD31^{bright}$ cells (arrowheads), and the $CD3^{+}$ cells within a $CD31^{bright}$ cell (arrows). **(A-C)** Nuclei stained with DAPI. Collapsed 9,45 µm-z-stack consisting of 21 consecutive images. Scale bars, 10 µm. **(D)** Orthoview CLSM image of collapsed z-stack shown in **(A)** demonstrating that $CD3^{+}$ T cells are internalized within the $CD31^{bright}$ cell building a cell-in-cell structure.
