# Supplementary material for: De novo Vessel Formation Through Cross-Talk of Blood-Derived Cells and Mesenchymal Stromal Cells in the Absence of Pre-existing Vascular Structures
Source: Front Bioeng Biotechnol. 2020 Nov 16;8:602210. doi: 10.3389/fbioe.2020.602210 (PMC7718010; doi:10.3389/fbioe.2020.602210)
Supplement: Supplementary file 1 [file Data_Sheet_1.zip › Supplemental Figure 4.PDF]

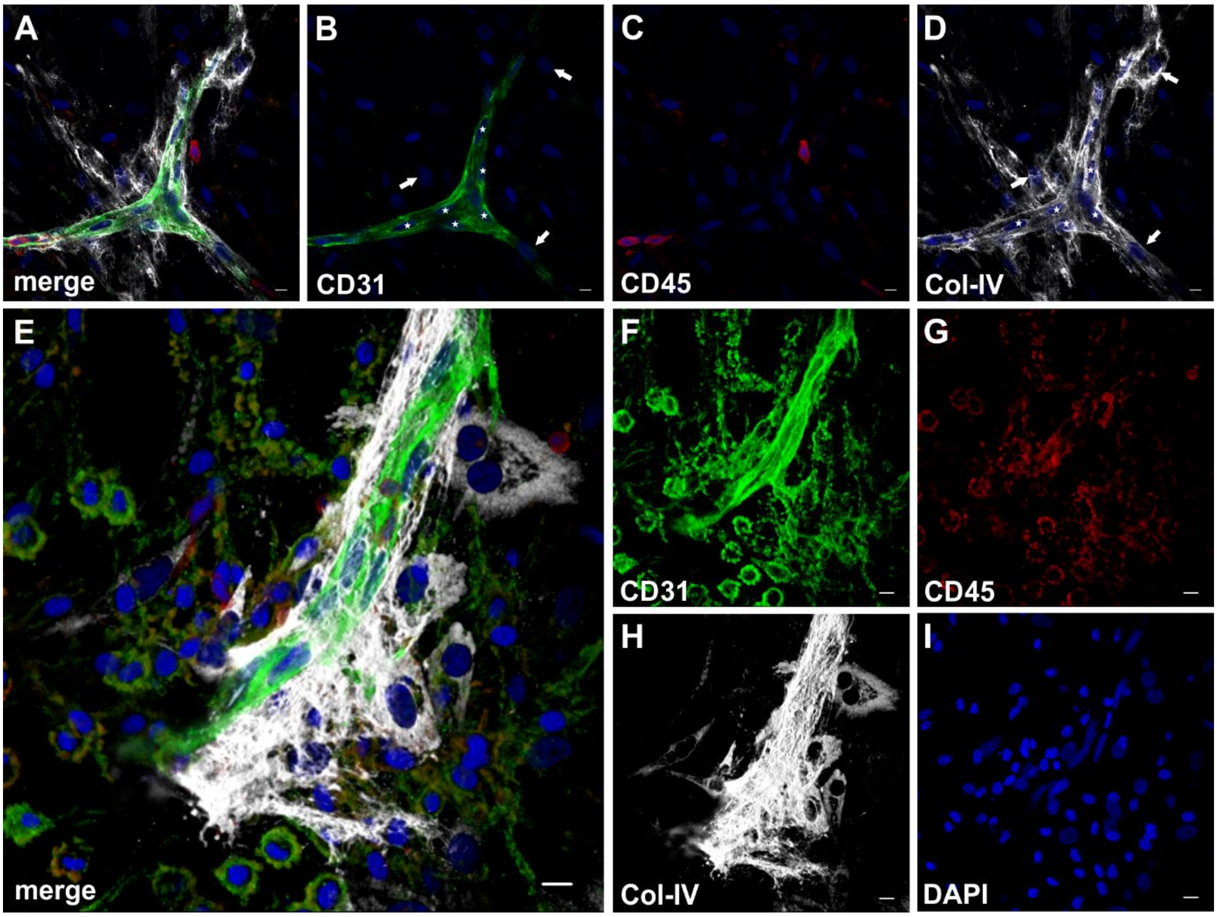

Supplemental Figure 4. **<u>Neo-vessels formed in co-cultures of MSC, enriched Mo and enriched T cells show similarities to vascular outgrowth from inflamed synovial tissue.</u>** **(A-D)** 2D cross-section images (frame 25/45) of collapsed z-stack images shown in Figure 6 **(A-E)** demonstrating that **(B)** $CD31^+$ EC of developing vascular structure co-express **(D)** Col-IV (stars in **(B)** and **(D)**). Perivascular stromal cells do not express **(B)** CD31 (arrows), but are **(D)** Col-IV^+ (arrows). **(C)** $CD45^+$ leukocytes are associated with vascular structure. **(A)** Merge. **(A-D)** Nuclei stained with DAPI. Scale bars, 10 µm. **(E-I)** Vascular outgrowth originating from inflamed synovial tissue fragment obtained from a patient with osteoarthritis, cultured in 3D fibrin matrix for 20 days as described in a previous publication (Rüger et al., 2018). **(F)** The $CD31^+$ vascular sprout is surrounded by **(H)** Col-IV matrix and by Col-IV^+ stromal cells that show close contact with perivascular cells co-expressing **(G)** CD45 and **(F)** CD31 consistent with macrophages. **(I)** DAPI stain. **(E)** Merge. **(E-I)** Collapsed 28 µm-z-stack consisting of 35 consecutive images. Scale bars, 10 µm.
