# Supplementary material for: De novo Vessel Formation Through Cross-Talk of Blood-Derived Cells and Mesenchymal Stromal Cells in the Absence of Pre-existing Vascular Structures
Source: Front Bioeng Biotechnol. 2020 Nov 16;8:602210. doi: 10.3389/fbioe.2020.602210 (PMC7718010; doi:10.3389/fbioe.2020.602210)
Supplement: Supplementary file 1 [file Data_Sheet_1.zip › Supplemental Figure 5.PDF]

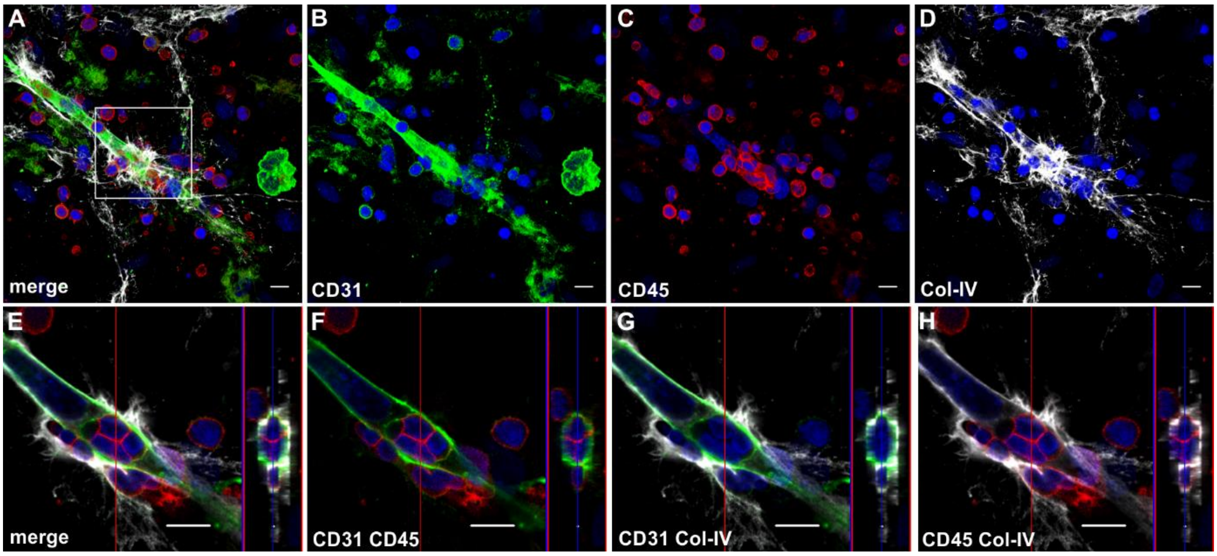


**Supplemental Figure 5.** **Neo-vessels develop in the absence of CD14$^+$ Mo.** Representative CLSM images of MSC co-cultured with Mo-depleted PBMC in 3D fibrin matrix on day 14 showing **(B)** a CD31$^+$ vascular structure associated with **(C)** numerous CD45$^+$ leukocytes. **(D)** Col-IV matrix is in alignment with the developing vessel. **(A)** Merge. **(A-D)** DAPI nuclear stain. Collapsed 13,5 µm-z-stack consisting of 30 consecutive images. Scale bars, 10 µm. **(E-H)** Orthoview CLSM images of collapsed z-stack shown in (**(A)**, boxed area). The 2D cross-section images (frame 16/30) show **(F)** four CD45$^+$ leukocytes inside of a CD31-lined vacuole within the CD31$^+$ elongated cell-in-cell structure. Tightly associated with the cell-in-cell structure is a **(G)** Col-IV$^+$ matrix lining resembling a basement membrane. **(H)** Neighboring CD45$^+$ leukocytes immediately adjacent to the CD31$^+$ cell-in-cell structure are also surrounded by a Col-IV$^+$ matrix. **(E)** Merge. **(E-H)** Nuclei stained with DAPI. Scale bars, 10 µm.
