# Supplementary material for: De novo Vessel Formation Through Cross-Talk of Blood-Derived Cells and Mesenchymal Stromal Cells in the Absence of Pre-existing Vascular Structures
Source: Front Bioeng Biotechnol. 2020 Nov 16;8:602210. doi: 10.3389/fbioe.2020.602210 (PMC7718010; doi:10.3389/fbioe.2020.602210)
Supplement: Supplementary file 1 [file Data_Sheet_1.zip › Supplementary Video Legends.DOCX]

**Video Legends**

**Video 1.** 3D co-culture MSC with PBMC

**Animated z-stack of developing vascular structure shown in Figure 1L**.The developing neo-vessel consists of CD31^+^ EC (green), is lined by Col-IV^+^ matrix (white) resembling a basement membrane and is surrounded by Col-IV^+^ stromal cells. CD45^+^ leukocytes (red) are closely associated with and aligned to the vascular cord. Two CD45^+^ cells are found within the developing vascular structure. Nuclear stain DAPI (blue). Animated 21,6 µm z-stack consisting of 54 consecutive images. Scale bar, 10µm.

**Video 2.** 3D co-culture MSC with enriched T cells

**Animated z-stack corresponding to Figure 5A.** Several CD45^+^ leukocytes (red) are closely associated with the bi-nucleated CD31^bright^ cell (green) in the center of the image. The CD31^bright^ cell does not express CD45. Col-IV^+^ stromal cells (white) are seen in the surrounding. Note the CD31^+^CD45^+^ cell in the upper left corner. Nuclear stain DAPI (blue). Animated 11,9 µm-z-stack consisting of 17 consecutive images. Scale bar, 10 µm.

**Video 3.** 3D co-culture MSC with enriched T cells

**Animated z-stack corresponding to Figure 5F**. Same as animated z-stack 2**,** but without CD31 staining, showing that the central bi-nucleated CD45^-^ cell (CD31^bright^ CD45^-^ cell seen in animated z-stack 2) is affiliated with Col-IV (white) deposited as fine granular network (white arrow). Note that the CD45^+^ cell (co-expressing CD31 in animated z-stack 2) in the upper left corner is also associated with a granular Col-IV matrix (red arrow). Nuclear stain DAPI (blue). Animated 11,9 µm-z-stack consisting of 17 consecutive images. Scale bar, 10 µm.

**Video 4.** 3D co-culture MSC with enriched T cells

**Animated z-stack corresponding to Figure 5G**. Two multi-nucleated CD31^bright^ cells (green) are surrounded by CD3^+^ T cells (red). Some CD3^+^ cells co-express CD31. One CD3^+^ cell is partly engulfed by a CD31^bright^ cell. Note the Col-IV expressing stromal cell on the left side of the two multi-nucleated CD31^bright^ cells, and the CD31^+^CD3^-^ mononuclear cells (white arrows) in the surrounding of the multinucleated CD31^bright^ cells. Nuclear stain DAPI (blue). Animated 17,28 µm-z-stack consisting of 36 consecutive images. Scale bar, 10 µm.

**Video 5.** 3D co-culture MSC with enriched T cells

**Animated z-stack corresponding to Figure 5L.** Same as animated z-stack 4**,** but without CD31 staining, showing that the two multi-nucleated cells in the center (seen as CD31^bright^ CD3^-^ cells in animated z-stack 4) are associated with a fine Col-IV^+^ granular network (white). Apart from the Col-IV expressing stromal cell on the left side, granular Col-IV staining is also seen around several CD3^-^ mononuclear cells and a number of CD3^+^ mononuclear cells in the surrounding of multi-nucleated cells. Nuclear stain DAPI (blue). Animated 17,28 µm-z-stack consisting of 36 consecutive images. Scale bar, 10 µm.

**Video 6.** 3D co-culture MSC with enriched T cells

**Animated z-stack corresponding to Figure 5M.** The multi-nucleated CD31^+^ cell (green) shows several internalized CD45^+^ cells (red) thereby building a cell-in-cell structure. Internalized CD45^+^ cells do not express CD31, and the multicellular CD31^+^ structure does not express CD45. The cell-in-cell structure is surrounded by Col-IV^+^ stromal cells and matrix. Nuclear stain DAPI (blue). Animated 9,9 µm-z-stack consisting of 22 consecutive images. Scale bars, 10 µm.

**Video 7.** 3D co-culture MSC with enriched T cells

**Animated z-stack corresponding to Figure 5O and P.** Same as animated z-stack 6**,** but without CD31 staining, showing a fine granular Col-IV network (white) around the multi-nuclear structure containing CD45^+^ cells (red). The cell-in-cell structure is surrounded by Col-IV^+^ stromal cells and matrix. Nuclear stain DAPI (blue). Animated 9,9 µm-z-stack consisting of 22 consecutive images. Scale bar, 10 µm.

**Video 8.** 3D co-culture of MSC, enriched Mo and enriched T cells

**Animated z-stack corresponding to Figure 6A.** CD31^+^ EC (green) of the developing neo-vessel co-express Col-IV (white). The branched vascular structure is aligned by Col-IV matrix (white) and associated with CD45^+^ leukocytes (red). Col-IV^+^ stromal cells localized to planes below the vascular structure are seen together with irregularly-shaped CD45^+^ leukocytes expressing intermediate levels of CD31 consistent with macrophages. Nuclear stain DAPI (blue). Animated 20,25 µm-z-stack consisting of 45 consecutive images. Scale bar, 10 µm.

**Video 9.** 3D co-culture of MSC, enriched Mo and enriched T cells

**Animated z-stack corresponding to Figure 6F.** CD45^+^ leukocytes (red) are localized inside of the developing vascular tube consisting of parietally located CD31^+^ EC (green) adjacent to Col-IV^+^ matrix (white). The neo-vascular structure shows two branch points. Nuclear stain DAPI (blue). Animated 16 µm-z-stack consisting of 40 consecutive images. Scale bar, 10 µm.

**Video 10.** 3D co-culture of MSC, enriched Mo and enriched T cells

**Animated z-stack corresponding to Figure 6K**. ‘Leading edge’ of a vascular structure. The vascular tube is surrounded by Col-IV matrix (white) and contains numerous CD45^+^ leukocytes (red), some of which show condensed nuclei (blue). CD31^+^ neo-vessel EC (green) show filopodial protrusions, especially the two distal cells at the tip of the vascular tube. One of the ‘tip cells’ is associated with several CD45^+^ leukocytes (red) present within a fine Col-IV network (white). Animated 9,75 µm-z-stack consisting of 25 consecutive images. Scale bar, 10 µm.

**Video 11.** 3D synovial explant culture vascular outgrowth

**Animated z-stack corresponding to Supplemental Figure 4E.** Vascular outgrowth originating from inflamed synovial tissue fragment obtained from a patient with osteoarthritis, cultured in fibrin matrix for 20 days according to Rüger et al., 2018. The CD31^+^ vascular sprout (green) shows similarities to vascular structures generated in 3D co-cultures of MSC with blood-derived mononuclear cells. The neo-vessel is surrounded by Col-IV matrix (white). Col-IV^+^ stromal cells (white) are in close contact with macrophage-like perivascular cells co-expressing CD45 (red) and CD31 (green). Nuclear stain DAPI (blue). Animated 28 µm-z-stack consisting of 35 consecutive images. Scale bar, 10 µm.

**Video 12.** 3D co-culture MSC with Mo-depleted PBMC

**Animated z-stack corresponding to Figure 7A.** The developing vascular structure shows parietally located CD31^+^ EC (green) and contains a CD45^+^ leukocyte (red) within a CD31-lined intracellular vacuole. Col-IV matrix (white) is tightly connected to and aligned with the nascent vessel, extending the ends of the CD31^+^ neo-vessel. Nuclear stain DAPI (blue). Animated 13,5 µm-z-stack consisting of 27 consecutive images. Scale bar, 20 µm.

**Video 13.** 3D co-culture MSC with Mo-depleted PBMC

**Animated z-stack corresponding to Supplemental Figure 5A.** The neo-vessel appears to develop via an elongated CD31^+^ cell-in-cell structure (green) showing intracellular CD31-lined vacuoles (white arrows), one of which contains four CD45^+^ leukocytes (red). The developing vascular structure is tightly connected to Col-IV matrix (white) covering the abluminal side of the neo-vessel. Note the CD31^bright^CD45^-^ bi-nucleated cell (green arrow). Nuclear stain DAPI (blue). Animated 13,5 µm-z-stack consisting of 30 consecutive images. Scale bar, 10 µm.
